# Protein Adsorption on Nano- and Microparticles: Dependence on Morphological and Physicochemical Properties of Particles and Effect on Particle–Cell Interactions

**DOI:** 10.3390/nano15131013

**Published:** 2025-07-01

**Authors:** Evgeniia Gerasimovich, Alexander Karaulov, Igor Nabiev, Alyona Sukhanova

**Affiliations:** 1 Laboratory of Nano-Bioengineering, National Research Nuclear University MEPhI (Moscow Engineering Physics Institute), 115409 Moscow, Russia; ewgenia-gerasimowitch@yandex.ru; 2 Department of Clinical Immunology and Allergology, Institute of Molecular Medicine, Sechenov First Moscow State Medical University (Sechenov University), 119146 Moscow, Russia; drkaraulov@mail.ru; 3 Université de Reims Champagne-Ardenne, BioSpectroscopie Translationnelle (BioSpecT)—UR 7506, 51100 Reims, France

**Keywords:** protein adsorption, nanoparticles, microparticles, protein corona, particle–cell interactions, drug delivery, targeting

## Abstract

Engineered nano- and microparticles are considered as promising tools in biomedical applications, such as imaging, sensing, and drug delivery. Protein adsorption on these particles in biological media is an important factor affecting their properties, cellular interactions, and biological fate. Understanding the parameters determining the efficiency and pattern of protein adsorption is crucial for the development of effective biocompatible particle-based applications. This review focuses on the influence of the morphological and physicochemical properties of particles on protein adsorption, including the pattern and amount of the adsorbed protein species, as well as the relative abundance of proteins with specific functions or physicochemical parameters. The effects of functionalization of the particle surface with polyethylene glycol, zwitterions, zwitterionic polymers, or proteins on the subsequent protein adsorption are analyzed. In addition, the dependences of protein adsorption on the protein species, biological buffers, fluids, tissues, and other experimental conditions are looked into. The influence of protein adsorption on the targeting efficiency of particle-based delivery systems is also discussed. Finally, the effect of the adsorbed protein corona on the interaction of the engineered micro- and nanoparticles with cells and the roles of specific proteins adsorbed on the particle surface in the recognition of the particles by the immune system are considered.

## 1. Introduction

Nanoparticles (NPs) and microparticles (MPs) of different types are used in a number of biomedical applications, including drug delivery [1,2], imaging [3], sensing [4,5], and theranostics [6]. In this connection, protein adsorption on NPs and MPs is of special interest because a so-called protein or biomolecular corona is known to form on particles entering biological media, endowing them with biological identity [7]. The corona alters the properties of the particles and largely determines their subsequent behavior and fate, including their blood clearance, cellular uptake, biodistribution, and toxicity and the immune response to them [8]. Therefore, a deeper understanding of the factors affecting protein adsorption can facilitate the rational design of particle-based biomedical applications.

The protein corona has been shown to form on a broad range of NPs and MPs, including gold [9,10], silica [11,12,13], poly(lactic-co-glycolic acid) (PLGA) [14,15,16], and superparamagnetic iron oxide NPs [17]; various polymeric particles, polymersomes, and nanogels [18,19,20]; and polystyrene (PS) [21,22,23], protein [24], and lipid [25,26,27] NPs. The interaction with proteins and, hence, the spectrum and amount of the adsorbed proteins, is shaped by the structural and surface properties of the particles, including their size, shape, morphology, structure, and stiffness, as well as the physicochemical characteristics of their surface, in particular, the hydrophobicity/hydrophilicity, charge, and functionalization. Experimental conditions, such as the incubation medium (protein solution, serum-containing culture medium, or pure serum or plasma), dispersion medium [28], and washing procedure [29], also influence protein–particle interactions. In turn, the composition of the adsorbed proteins may affect both the biological response to the particles, including the cellular uptake and blood clearance rates, and the particle toxicity and targeting efficiency. A variety of approaches have been developed to reduce protein adsorption or change its pattern: surface modification with polyethylene glycol (PEG), its derivatives, and zwitterionic compounds; precoating particles with specific proteins; and changing the surface chemistry so that the particles bind desirable proteins.

The protein corona is generally considered to comprise two layers: a “hard” corona consisting of tightly bound proteins and a “soft” corona formed by loosely bound proteins that are dynamically exchanged with the proteins in the surrounding medium [30]. Most studies deal with the composition and effects of the “hard” corona. The isolation techniques, e.g., centrifugation or magnetic separation, and the number of washes may dramatically affect the results of protein adsorption studies [31]. Centrifugation is the most frequently used technique due to its availability and high throughput; however, it can cause particle aggregation and change protein corona composition [32]. Magnetic separation is an effective technique, but its use is limited. In situ analysis of the protein corona extensively employs field-flow fractionation techniques, which offer substantial advantages: a high efficiency of particle separation, versatility, minimal sample disturbance, and compatibility with mass spectrometry [33]. The methods of analysis of the isolated corona include SDS electrophoresis, mass spectrometry, atomic force microscopy, transmission electron microscopy, and protein quantification assays [32]. Regarding qualitative analysis of the protein corona, SDS electrophoresis offers relatively fast analysis of the corona composition based on the molecular weights of the proteins and is widely used for initial screening. For in-depth analysis, mass spectrometry is more suitable because it allows protein identification with a high sensitivity, but higher cost and complexity limit its use. On the other hand, isolation and identification of “soft” corona proteins may be unreliable because of the dynamic nature of the protein corona, weak interactions between its components, and limitations of the separation methods. A combination of advanced separation and analytical techniques (in situ click-chemistry reactions and mass spectrometry) allowed the detection of the “soft” corona proteins on silica and PS particles [34]. The proteins identified in the “soft” corona were also found in the “hard” corona, although they presumably differed in their binding strength. In this study, a significant role of weakly bound proteins in particle–cell interactions was demonstrated. In another study, the fishing method based on bio-layer interferometry coupled with LC-MS/MS was used for characterization of the proteins of the “hard” and “soft” coronas formed on chiral NPs [35]. The time-dependent protein composition of the “soft” corona was found to correlate with blood clearance and biodistribution. In situ laser confocal microscopy combined with microfluidics was used to detect three steps of protein adsorption forming three protein layers: proteins irreversibly bound to the particle surface, proteins irreversibly bound to the previous layer of proteins (the “hard” corona), and reversibly bound proteins (the “soft” corona) [36]. In the case of MPs with a low-fouling zwitterionic coating, only the “soft” corona was formed.

The formation of the protein corona on particles is considered to be a key obstacle to clinical use of nanomedicines, including drug delivery systems, because it can lead to a reduction in blood circulation time, the activation of the immune response, a loss of targeting ability, and a decrease in the drug release rate [8]. However, there are examples of improvement of the targeted delivery of NPs upon protein adsorption on them [37]. In addition, analysis of the protein corona formed on NPs in human systemic circulation provides an opportunity to detect low-molecular-weight and low-abundance biomolecules [38]. The composition of the adsorbed proteins varies not only between individual donors, but also between diseases, which offers the opportunity to enhance the sensitivity and specificity of diagnosis [39].

In this review, we analyze the dependence of protein adsorption on the morphological and physicochemical properties of the particles. In addition, different approaches to surface modification reducing protein adsorption are discussed, along with strategies for using the protein corona to improve the biological performance of the particles. Finally, we consider how protein adsorption onto engineered particles affects their interactions with living cells, with a special focus on their uptake by immune and other cells and the specific binding of functionalized particles with target cells.

## 2. Dependence of Protein Adsorption on Particle Morphology

### 2.1. Size and Shape

**Figure 1:**
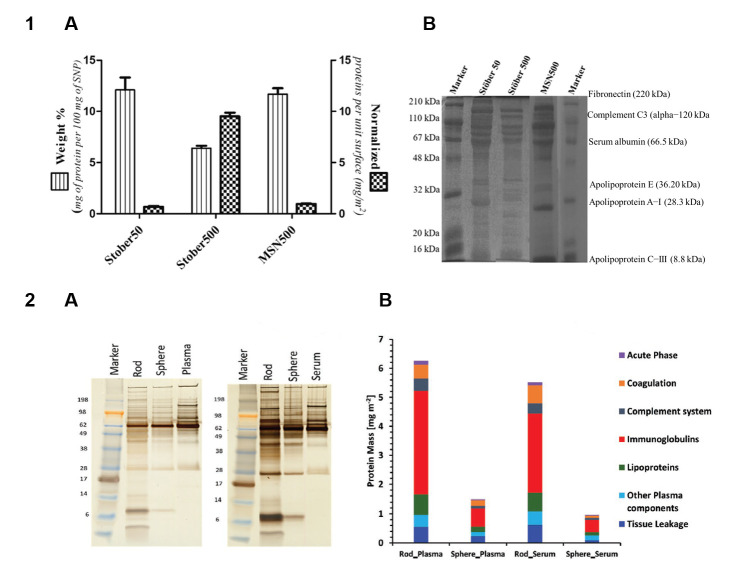
Dependence of protein adsorption on the morphological properties of particles. **Panel 1.** The effect of size and porosity of silica particles on protein adsorption. (**A**) The amount of protein recovered from each nanoparticle by BCA assay. (**B**) SDS-PAGE of the adsorbed proteins recovered from the hard corona; the intensity of the band indicates the abundance of the protein recovered. The proteins listed are for reference. Abbreviations: SNP, silica nanoparticle; MSN, mesoporous silica nanoparticle. Adapted with permission from Ref. [12]. **Panel 2.** The effect of the shape of silica particles on protein adsorption. (**A**) Gel electrophoresis of proteins adsorbed on rod-shaped and spherical particles. (**B**) Quantification of the adsorption of human plasma and serum proteins to the particles and classification of proteins identified by quantitative LC-MS according to their biological functions. Adapted with permission from Ref. [48].

The size of NPs and MPs is the parameter most thoroughly studied in terms of its effect on protein adsorption on the particles. Regarding NPs, it is generally assumed that smaller ones, whose surface is more curved, adsorb proteins more weakly because of steric effects [40]. Speaking in terms of the amount of proteins adsorbed per unit weight of particle, this is counterbalanced by smaller particles having a larger relative surface area. The latter effect has been shown to be prevailing; e.g., smaller chitosan NPs adsorb more bovine serum protein (BSA) per unit weight [41]. However, the amount of adsorbed proteins normalized by surface area rises with increasing particle size, which has been shown for gold, PS, silica, elastin-like polypeptide (ELP), and solid lipid NPs [12,25,42,43,44,45,46] (Figure 1, Panel 1). This is also true for the adsorption of high-density lipoproteins (HDLs) [47].

The size of engineered particles affects not only the quantity of adsorbed proteins, but also the composition of the protein corona. An early study showed that protein coronas formed on 50 and 100 nm neutral PS NPs in human plasma had similar compositions (with an ~80% coincidence rate), but the differences were greater in the cases of amino- and carboxyl-modified NPs [21]. This study also demonstrated a complex relationship between the protein corona composition and other characteristics of the particles. However, the spectra of proteins that silica NPs adsorbed from soluble yeast protein extracts were similar for NPs of three different sizes [46], and 50 and 500 nm silica NPs incubated in fetal blood serum (FBS)-supplemented RPMI medium were also reported to have about the same protein corona composition [12] (Figure 1, Panel 1). Another study, however, found that smaller silica NPs tended to adsorb lower-molecular-weight proteins, probably due to their higher surface curvature [44]. An analysis of the adsorption of plasma proteins on ELP nanoparticles showed that three major proteins (albumin, IgG, and complement factor 3) were adsorbed on all types of particles, whereas the profile of other proteins varied. ELP NPs of different sizes were also found to inhibit blood clotting, the effect of smaller NPs being stronger [45]. The discrepancies between the data on the protein coronas on particles with different sizes can be explained by variations in the particle composition and surface chemistry, as well as differences in protein sources (e.g., plasma, yeast extract, or FBS). Differences between the analytical approaches used to assess the protein corona composition are also an important factor that can contribute to inconsistencies in the reported results.

The effect of adsorption on the protein conformation also depends on the particle size. Specifically, experiments on the adsorption of BSA and myoglobin onto 30 to 1000 nm particles showed that only those larger than 200 nm caused conformational changes in the adsorbed proteins [49]. Similarly, PS particles of different sizes had different effects on the formation of amyloid fibrils from hen egg-white lysozyme [50].

The effect of the particle shape on the adsorption of serum proteins was studied using spherical, rod-shaped, and faceted silica particles [48]. The spherical particles formed a more homogeneous corona with a higher albumin content. The protein coverage and corona composition of the faceted particles were found to strongly depend on the number of exposed facets and the aspect ratio. In another study, rod-shaped silica particles adsorbed three and four times more protein from plasma and serum, respectively, than spherical ones, but the protein compositions were similar [51] (Figure 1, Panel 2). A comparison of protein adsorption on gold NPs of four shapes (spheres, rods, stars, and cages) with three surface modifications (methoxy, carboxy, and amino groups) showed that the cage-shaped NPs adsorbed the smallest amount of protein, and the nanostars, the largest amount [10]. The compositions of the protein coronas formed on the nanostars and nanospheres were similar to each other but considerably differed from that of the corona on nanocages. Interestingly, the particle shape more strongly affected the composition of the protein corona than the surface functional groups did. In vivo experiments with gold nanostars and nanorods of different sizes confirmed that the composition of the protein corona formed on them depended on both the size and shape of the NPs [9].

### 2.2. Surface Morphology

The surface morphology of particles (e.g., porosity) considerably affects protein adsorption. A smoother surface tends to adsorb more protein per unit area than a porous one, although the amount of proteins absorbed per porous particle is still larger compared to a smooth particle of the same size due to the considerably increased relative surface area [12]. On the other hand, porous silica particles were shown to adsorb larger amounts of low-molecular-weight proteins than dense particles, which was attributed to the size-exclusion effect [44]. More detailed investigation of the effect of pore size on protein adsorption revealed a nonlinear dependence, which led to the conclusion of a critical pore size for each particular protein at which the adsorption capacity is significantly enhanced [52].

Etching seems to decrease the protein absorption capacity of particles. For example, PS MPs with an etched surface absorbed less BSA and fibrinogen compared to nonetched MPs [22].

**Figure 2:**
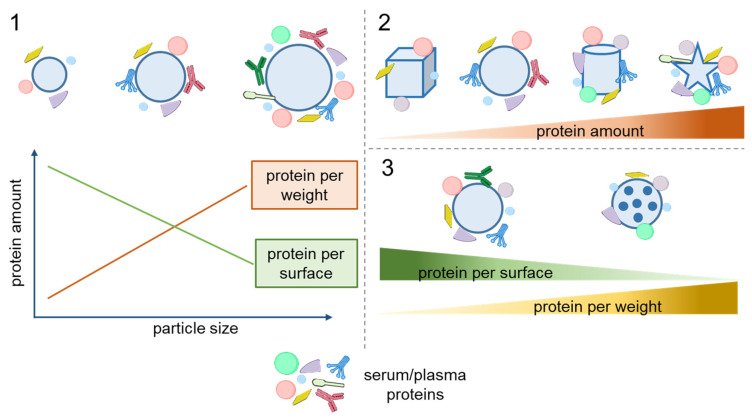
General patterns of protein adsorption on particles with different (**1**) sizes, (**2**) shapes, and (**3**) surface morphologies.

Thus, the particle size, shape, and surface morphology can affect both the quantity and composition of the adsorbed proteins. Understanding the effects of particle morphology is crucial in designing tailor-made nano- and microcarriers for biomedical applications. The general patterns of influence of these parameters on protein adsorption are summarized in Figure 2.

### 2.3. Structure

Both the amount and composition of proteins adsorbed vary depending on the particle structure. For example, core/shell polymeric MPs and hollow MPs lacking the core (capsules) differ in the protein corona [53]. Both types of microstructures functionalized with monoclonal antibodies retained the targeting capacity if they had an additional protein layer on their surface; however, the relative number of cells targeted by the core/shell particles was larger than those targeted by the capsules (90% and 70%, respectively). In addition, the capsules adsorb a considerably larger total amount of proteins from human serum and plasma than the core/shell particles [54]. The composition of the protein corona also varied between the two types of microstructures: core/shell MPs tended to bind a larger amount of apolipoproteins, whereas the capsules adsorbed relatively more complement factors and immunoglobulins.

Thus, the MP structure is also an important factor affecting their protein corona and, hence, their interactions with live cells.

## 3. Dependence of Protein Adsorption on Physicochemical Properties of Particles

### 3.1. Composition

The chemical composition of NPs and MPs largely determines their interaction with proteins. Methacrylic acid co-methyl methacrylate (MAA) and polymethyl methacrylate (PMMA) MPs incubated in human serum and plasma displayed different protein adsorption profiles, differing, in particular, in the affinity to complement components, which was related to weaker complement activation by MAA MPs [55]. Three types of alginate MPs, sulfated alginate, high G alginate, and poly-L-lysine-coated alginate ones, also adsorbed different amounts of complement components and coagulation factors, which were correlated with their different inflammatory and fibrotic profiles [56].

**Figure 3:**
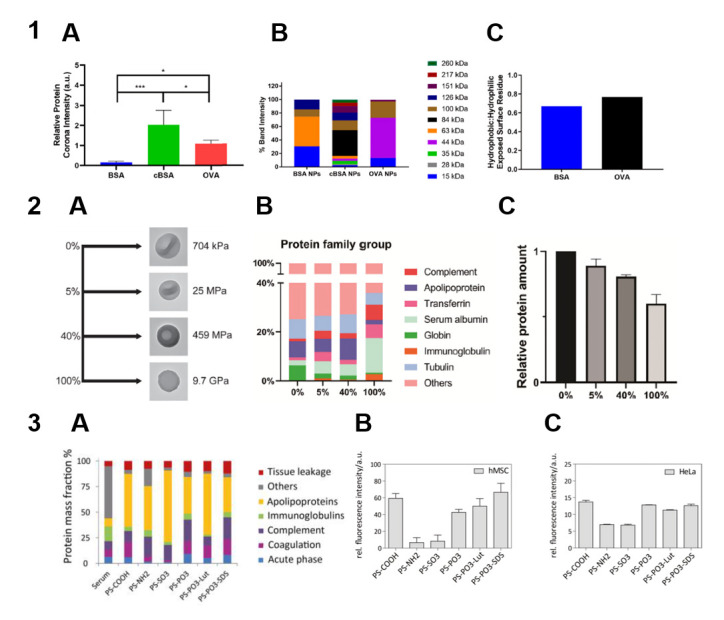
Dependence of protein adsorption on the physicochemical properties of particles. **Panel 1**. The effect of the nanoparticle composition and charge on protein adsorption. The protein coronas were analyzed by gel electrophoresis and nanoliquid chromatography–mass spectrometry. (**A**) Relative intensities of the adsorbed protein coronas on albumin, cationic albumin, and ovalbumin cross-linked nanoparticles. The average intensities were measured using densitometry and compared against the average intensity of the protein ladder in each image (*n* = 4; * *p* < 0.05, *** *p* < 0.001; one-way ANOVA). (**B**) Average distribution of the molecular weight bands identified in the nanoparticle protein coronas (*n* = 4). (**C**) The ratios between the exposed hydrophobic and hydrophilic residues in albumin and ovalbumin. Abbreviations: BSA, bovine serum albumin; cBSA, cationic BSA; OVA, ovalbumin. Adapted with permission from Ref. [24]. **Panel 2**. The effect of particle stiffness on protein adsorption. (**A**) Types of silica nanoparticles with different *tetraethyl orthosilicate* (TEOS)*-to-triethoxyvinyl silane* (TEVS) ratios (**0%** TEOS/100% TEVS, **5%** TEOS/95% TEVS, **40%** TEOS/60% TEVS, and **100%** TEOS/0% TEVS) and their stiffnesses (Young moduli). (**B**) The results of liquid chromatography–mass spectrometry analysis of silica capsules incubated with FBS for 3 h, with protein family group coverage up to 50%. (**C**) Relative total protein amounts in different groups of the nanoparticles, with the highest protein amount taken to be 1. The mean ± SD values from three independent replicates are shown. Abbreviation: FBS, fetal blood serum. Adapted with permission from Ref. [58]. **Panel 3**. The effect of surface functionalization on protein adsorption. (**A**) Distributions of the seven major protein groups in human serum and in the protein coronas of differently functionalized polystyrene nanoparticles presented as protein mass fractions (%). (**B**) Nanoparticle uptake by human mesenchymal stem cells quantitatively assessed by flow cytometry. (**C**) Nanoparticle uptake by HeLa cells quantitatively assessed by flow cytometry. Abbreviations: PS, polystyrene; Lut, Lutensol AT50; SDS, sodium dodecyl sulfate. Adapted with permission from Ref. [23].

Hydrophobic interactions are among the main driving forces of protein adsorption [57]. Therefore, the hydrophobicity/hydrophilicity of the particle material is a major factor in protein–particle interactions. For example, differences in composition between the protein coronas formed on PLGA, cholesterol, and hybrid PLGA–cholesterol NPs were explained by their differences in hydrophobicity [15]. Engineered ovalbumin NPs adsorbed more protein than BSA NPs, which correlated with a higher hydrophobicity of the surface of ovalbumin NPs [24] (Figure 3, Panel 1). The higher affinity of serum proteins to more hydrophobic particles leading to differences in protein corona composition was confirmed in a study on poly(ε-caprolactone) (PCL) and PLGA nanoparticles [16]. This also agrees with the finding that poly(methyl methacrylate-*co*-styrene) particles bound more BSA, IgG, and fibronectin the higher their styrene content was [19].

Differences between the phospholipid compositions of lipid nanodiscs were found to determine variations in protein adsorption capacity and affinity to apolipoprotein E (ApoE) [26]. Nanodiscs with a higher capacity for ApoE exhibited better brain targeting in mice, which agrees with previous data [59]. In addition, slight changes in complement activation were detected for these lipid NPs, which influenced particle stability.

There is evidence that the NP electric charge is a major factor in the effect of the composition of lipid NPs on the formation of their protein coronas. NPs containing negatively charged lipids were reported to form an apolipoprotein-rich corona, which facilitated their delivery to tumors compared with NPs that had a vitronectin-rich corona [27].

In the case of polymeric NPs and MPs, the molecular weights of their component may be another aspect of the relationship between the particle composition and the formation of the protein corona. For example, high-molecular-weight chitosan NPs bound less protein than low-molecular-weight NPs with a size of about 100 nm [34]. This was explained by a higher structural rigidity of the higher-molecular-weight polymer, which agrees with the data on protein adsorption from an FBS-supplemented cell medium onto MPs consisting of elastomers with different molecular weights and, correspondingly, molecular chain flexibilities [60].

Thus, the composition of particles can affect the amount and profile of biologically relevant proteins adsorbed on their surface and, hence, the physiological response to them. In some cases, the enrichment of particles with specific proteins can improve their targeting properties.

### 3.2. Stiffness

Detailed analysis of the formation of the protein corona on silica NPs differing in stiffness (with the Young modulus ranging from 704 kPa to 9.7 GPa) showed that the amount of adsorbed proteins decreased with increasing stiffness [58]. The profile of adsorbed proteins varied between these NPs, with higher amounts of complement factor C3 and immunoglobulins adsorbed on stiffer particles, which correlated with elevated macrophage uptake (Figure 3, Panel 2). Similarly, hydrogel NPs with Young moduli ranging from 45 kPa to 760 MPa coated with a PEGylated lipid bilayer differed in the composition of the protein corona formed upon incubation with mouse plasma [61]. Apolipoprotein A-I was identified as a protein whose proportion in the protein corona was correlated with the blood clearance rate. This pattern was retained if the composition of the NP outer shell was changed, which suggests that the particle stiffness affects the protein adsorption more strongly than the particle composition. However, studies comparing the effects of stiffness and other characteristics of the particles are limited. Surface chemistry is considered as one of the key factors in protein corona formation, as discussed below.

### 3.3. Surface Chemistry

In a pioneering study on protein adsorption onto PS particles of two sizes with unmodified, carboxyl-modified, and amino-modified surfaces [21], both size and surface modification were found to affect the formation of the protein corona. An analysis of protein adsorption from human serum onto PS particles with different surface functional groups (–COOH, –NH_2_, –SO_3_, and –PO_3_) [23] showed that the PS–COOH particles adsorbed twice as many protein molecules as the other types of PS NPs. All types of particles predominantly adsorbed apolipoproteins and complement factors, the particles functionalized with –NH_2_ and –SO_3_ exhibiting the highest enrichment with these substances, as well as the weakest cellular uptake. The abundance of absorbed ApoH was the highest on the surface of PS–COOH and PS–PO_3_ particles, whose cellular uptake rate was also the highest (Figure 3, Panel 3). Later, protein adsorption on the PS–COOH and PS–NH_2_ surfaces was studied upon incubation in a serum-containing cell culture medium [62]. The surface functionalized with –COOH favored the adsorption of proteins involved in integrin signaling, which promoted an anti-inflammatory response from macrophages. The roles of the amino and hydroxyl groups on the NP surface in the formation and evolution of the protein corona were further studied in experiments on nanovesicles coated with different amino-modified glycosylated polyhydroxy polymers to obtain different amino-to-hydroxyl-group ratios on the NP surface [63]. The NPs with the maximum NH_2_-to-OH ratio absorbed the smallest amounts of immunoglobulin, which prolonged their blood circulation, and the largest amounts of CD44 and osteopontin, which promoted their internalization by cancer cells.

Protein adsorption on poly(styrene)-block-poly(ethylene glycol) (PS-*b*-PEG) NPs was significantly affected by the introduction of cationic or anionic groups into the surface, although the resulting shift of their ζ-potential was small [64]. Functionalization with the cationic polymer poly(N,N-dimethylaminoethyl methacrylate) with a 1% PS-*b*-PEG substitution enhanced protein adsorption on the NPs and facilitated their nonspecific cell binding, whereas the NPs modified with the anionic poly(acrylic acid) (PAA) displayed weak protein adsorption and specific binding to macrophages.

Comparison of protein adsorption on particles consisting of BSA and cationic BSA (with carboxyl groups converted into primary amines) showed that the cationic particles bound significantly more proteins from FBS [24]. The protein coronas of the two types of particles distinctly differed, the corona of the cationic BSA NPs being more diverse. However, both BSA and cationic BSA NPs were readily taken up by macrophages.

The roles of acidic and ester surface groups rendering the particle surface hydrophilic and hydrophobic, respectively, in the adsorption of human serum and plasma proteins on NPs were analyzed using PLA and PLGA particles [65]. It was found that some proteins preferentially bound to hydrophobic or hydrophilic NPs, the total amount of adsorbed proteins increasing with an increase in the number of functional groups on the particle surface.

The effects of particle surface charge and hydrophilicity/hydrophobicity on the adsorption of proteins from human plasma were estimated using three types of colloidal NPs (soft core/multishell NPs and cationic (Eudragit RS) and anionic (ethyl cellulose) rigid NPs) and three types of nanogels (thermoresponsive dendritic polyglycerol and two amino-functionalized nanogels) [20]. The cationic and anionic rigid NPs adsorbed the greatest amounts of proteins from human plasma due to their high surface charges. These NPs also had the most variable corona and were characterized by low cellular uptake and drug release rates. The core/multishell NPs with a neutral charge absorbed small amounts of albumin, but the presence of the protein corona still impaired their cellular uptake. The high hydrophilicity of all the nanogels interfered with protein adsorption on them, their protein corona being too sparse to affect their uptake by primary human macrophages, but cytokine release from them was enhanced.

However, in the case of silica particles, the distribution of surface silanol (–SiOH) groups affected BSA adsorption more strongly than the surface charge did [66].

The formation of the protein corona on the surface of nonfunctionalized particles and those functionalized with –COOH and –NH_2_ groups with sizes of 50, 100, 200, 500, and 1000 nm was studied using an in vitro model of gastrointestinal digestion with subsequent incubation in serum-containing cell culture medium [67]. The in vitro digestion increased the macrophage uptake of neutral 50, 100, and 200 nm particles, but not neutral 500 or 1000 nm NPs or charged 100 nm particles. Liquid chromatography–tandem mass spectrometry (LC-MS/MS) showed that the digestion affected the composition of the protein corona formed upon incubation with serum, the presence of specific proteins (complement and coagulation cascade proteins) in the corona being correlated with the intensity of cellular uptake of the particles. This study showed that both the charge and size of particles determined their interaction with proteins, thus affecting their biological interaction, which agrees with earlier data [21]. A recent study demonstrated that nanoparticle size and charge determined the pattern of adsorption of saliva proteins [68].

**Figure 4:**
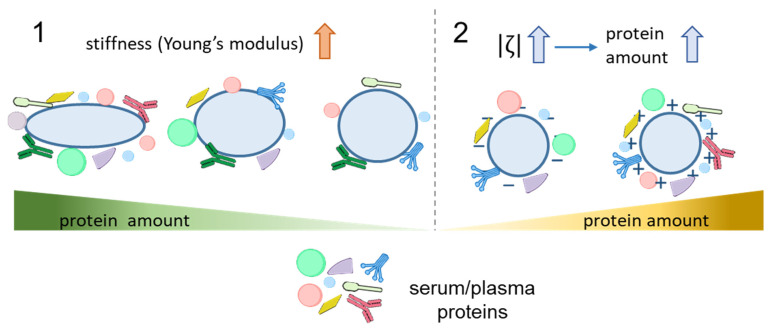
General patterns of protein adsorption on particles with different (**1**) stiffnesses and (**2**) surface charges.

Overall, surface properties play an important role in determining the interaction of particles with proteins. In general, particles with a higher surface charge adsorb more proteins. Surface cationic groups also tend to more effectively promote protein binding. Irrespective of the surface charge, some functional groups (e.g., amino, hydroxyl, and carboxyl ones) have turned out to be important factors in the adsorption of specific proteins, thereby determining the biological response to the particles. The influence of physicochemical parameters on protein adsorption onto particles is summarized in Figure 4 and Table 1.

## 4. Surface Modifications Affecting Protein Adsorption on Particles

Various surface modifications are used to control the protein adsorption on NPs and MPs. The modification of particle surfaces with hydrophilic polymers or zwitterions is typically used to reduce the amount of adsorbed proteins (antifouling or protein-repellent surfaces) and avoid nonspecific cellular uptake, imparting the particles with so-called stealth properties. Precoating of particles with certain proteins or protein mixtures allows altering further protein adsorption from biological media and modifying the cytotoxicity and cellular uptake of the particles. Another type of surface modification is used to enhance the adsorption of specific proteins in order to obtain particles with desirable biological properties, such as biodistribution parameters and specific binding. The results of recent studies on different particle modifications and their effects on protein adsorption are summarized in Table 2.

### 4.1. Surface Modification with Hydrophilic Polymers

Polyethylene glycol is widely used for particle surface modification due to its hydrophilicity and biocompatibility, as well as because it increases the colloidal stability and blood circulation time of the particles. PEGylation has recently been reported to mitigate protein adsorption on various particles, such as PLGA [78], layered double hydroxide (LDH) [71], silica [73,75,79], strontium sulfite [77], hydroxyethyl starch (HES) [82,83], and gold [43,84] NPs. Various PEG derivatives are also used for this purpose. For example, a copolymer consisting of polyethylene glycol and allyl glycidyl ether blocks (PEG-*b*-AGE) has been used for coating iron oxide NPs [79]; the PEG-based surfactant Lutensol AT50, for coating PS NPs [70]; and chitosan-g-methoxy poly(ethylene glycol), for surface modification of hydrogel microcapsules [81]. Apart from the protein-repelling effect, PEGylated particles are also characterized by a higher colloidal stability [71,82,85] and a lower nonspecific cellular uptake rate [69,85].

PEGylation not only reduces the amount of proteins adsorbed from biological media, but also affects the composition of the protein corona. PEG- and poly(ethyl ethylene phosphate) (PEEP)-coated PS nanocarriers were shown to form protein coronas enriched with apolipoproteins, such as ApoA1 and clusterin (apolipoprotein J, ApoJ) [80]. A high clusterin content of the protein corona derived from plasma was found to be associated with a weak uptake of PEG- and PEEP-modified nanocarriers by RAW264.7 macrophages. Experiments on the cellular uptake of clusterin-coated PEG-NPs confirmed the predominant role of this protein in suppressing particle–cell interactions.

PEGylation of 100 and 200 nm PLGA particles decreased the abundance of proteins related to immune response [78].

The functionalization of strontium sulfite NPs with biotinylated PEG was reported to increase the proportion of albumin and reduce the overall diversity of adsorbed proteins in the protein corona [77].

The molecular weights and lengths of the PEG chains used for the modification of NPs directly affect the amount of adsorbed proteins. For instance, the adsorption rate and the final quantity of proteins adsorbed on PEGylated gold NPs increased as the PEG molecular weight increased in the sequence 5000 < 10,000 < 30,000 Da [76]. The PEG molecular weight also affected the composition of the protein corona on PEG-coated gold NPs [72]. The relative abundances of albumin and transferrin were higher on the surface of NPs modified with 550 Da PEG compared with those modified with 350 and 1000 Da PEGs. Serum albumin is known to act as a dysopsonin, whereas transferrin can facilitate the binding of NPs with various cancer cells overexpressing the Tf receptor. Correspondingly, doxorubicin-conjugated NPs modified with 550 Da PEG were the most cytotoxic for TfR-expressing HepG2 cells and exhibited the best tumor targeting in mice among all formulations tested [72].

The conformation of PEG (brush or mushroom) [131] is determined by both the molecular weight and the grafting density of PEG on the particle surface. This factor also influences protein adsorption and subsequent cellular uptake. It was shown that the modification of ovalbumin NPs with PEG in the brush conformation led to a decrease in serum albumin adsorption and an increase in clusterin adsorption, which weakened the NP uptake by macrophages [69]. The effect of PEG modification on protein adsorption and particle interaction with cells varies between different types of particles. For example, the protein coronas formed on PEGylated mesoporous silica particles and PEGylated liposomes in human blood plasmas from different individual donors considerably differed in composition, which determined differences in their interaction with immune cells. In contrast, PEG-only capsules (obtained by removing the core particle after PEGylation) interacted weakly if at all with immune cells, irrespective of the plasma donor [74].

The effect of particle PEGylation on protein adsorption can be used to improve the targeting ability of the particles. For example, PEGylated gold NPs conjugated with Herceptin retained the capacity for specifically binding with targets after incubation with serum, whereas NPs without PEG coating lost this capacity [84]. PEGylation followed by functionalization with targeting ligands can provide an efficient combination of “stealth” behavior (reduction in nonspecific interaction with plasma proteins) and targeting. This has been confirmed in a study on PEGylated hydroxyethyl starch (HES) nanocarriers conjugated to mannose, which retained the capacity for binding with dendritic cells after incubation in human plasma [82]. Moreover, the protein corona formed on Onyvide, a PEGylated liposomal drug, promoted the uptake of the drug by target cancer cells [85].

However, a serious drawback of PEGylated nanocarriers is that their administration may provoke an immune response leading to their rapid elimination from the body [132]. The results of experiments on mice suggest that this is mediated by the alteration of the composition of the protein corona formed on the injected particles in the presence of anti-PEG IgM antibodies, presumably due to the activation of the complement cascade [133]. The immune response to PEGylated drug carriers can substantially reduce the drug efficacy and cause hypersensitivity reactions and other side effects [134]. This calls for the search for alternatives to PEG.

Hyperbranched polyglycerol (PG), a non-immunogenic biocompatible hydrophilic polymer, is considered as a PEG alternative [134]. PG-modified NPs of various types and sizes have proved to be more resistant to protein adsorption than their PEG-modified counterparts. PG-modified particles almost completely evaded macrophage uptake upon incubation in 55% serum or cell culture medium supplemented with 10% FBS [88]. However, this is not true for all types of nanocarriers. PG modification of liposomes, conversely, enhanced their uptake by macrophages, whereas PEG modification decreased the uptake rate [87].

Poly(phosphoester) was used for the modification of PS NPs as a biodegradable alternative to PEG. This resulted in reduced protein adsorption and decreased macrophage uptake upon incubation with plasma [89].

The modification of NPs with poly(2-ethyl-2-oxazoline) efficiently reduced protein adsorption on them and their nonspecific cellular uptake [90].

Thus, PEGylation is a well-studied polymer modification of various types of particles that suppresses protein adsorption and alters the composition of the protein corona, resulting in a reduced nonspecific cellular uptake of the particles. However, the biomedical use of this approach has some limitations, including toxicity and the formation of anti-PEG antibodies. Several non-immunogenic and low-toxic polymers have been tested as alternatives to PEG and exhibited a comparable efficiency in the stealth modification of particles.

### 4.2. Surface Modification with Zwitterionic Polymers

Zwitterionic polymers have gained much attention as an alternative to antifouling PEG coating in biomedical applications, mainly because they tend to form a hydration layer on the particle surface [135]. In addition, zwitterions are low-toxic, do not induce inflammation or antibody production, and have a very low hemolytic activity and relatively long circulation half-time (40 h in mice) [136]. Zwitterionic compounds include low-molecular-weight zwitterions, such as amino acids, sulfobetaine and carboxybetaine derivatives, and polymeric zwitterionic materials. The latter can be subdivided into polymers in which each monomer carries both positive and negative charges, such as poly(sulfobetaine), poly(2-methacryloyloxylethyl phosphorylcholine), and poly(sulfobetaine methacrylate), and mixtures of positively and negatively charged monomers [137].

Cysteine and other amino acids can be used as low-molecular-weight zwitterionic ligands. Experiments using silica NPs modified with cysteine as a zwitterion and biotin as a model targeting ligand demonstrated a reduction in protein adsorption and effective targeting of NPs after incubation in 10% or 50% human plasma [91]. Cysteine, lysine, and arginine functionalization of silica NPs were shown to make these NPs resistant to protein adsorption from a BSA solution and FBS [92]. Another low-molecular-weight zwitterion, organosiloxane, used for the modification of silica particles, inhibited protein adsorption on these particles and ensured their high stability [93].

Recently, zwitterionic peptides with various amino acid sequences and compositions were used to modify gold NPs. They were found to be promising antifouling surface functionalities [94]. The protein adsorption pattern was found to depend more on the charge motif and sequence than on the amino acid composition, and it influenced the rate of NP uptake by macrophages.

Further development of this approach led to the use of bi-functionalized zwitterionic/–COOH silica NPs combining antifouling properties with the availability of functional groups suitable for conjugation with biomolecules [95]. Magnetic silica NPs functionalized with amino and alkene functional groups along with the betaine zwitterion exhibited reduced adsorption of BSA, lysozyme, and FBS while retaining the capacity for interacting with biomolecules [97]. However, double functionalization of silica NPs with a zwitterion (sulfobetaine) and amino, mercapto, or carboxylic functional groups imparted antifouling properties and hindered the interaction between the functional groups and cells [99].

**Figure 5:**
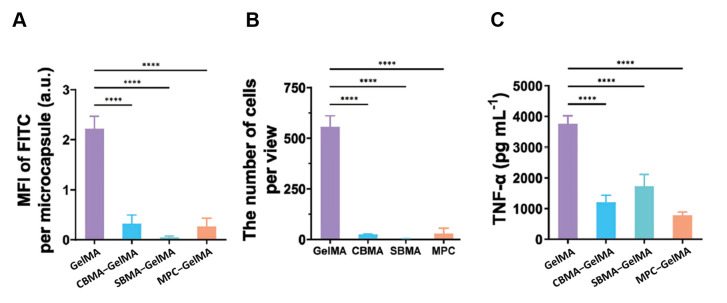
Protein adsorption on zwitterion-coated microcapsules and interaction of the microcapsules with cells. (**A**) The intensities of fluorescence of different microcapsules incubated with fluorescein isothiocyanate-labeled bovine serum albumin (BSA–FITC); data are presented as the mean ± SD, *n* = 10. (**B**) The numbers of NIH/3T3 cells stained with 4′,6-diamidino-2-phenylindole (DAPI) adhering to different hydrogels. (**C**) The level of TNF-α secreted from macrophages after culturing in the presence of different microcapsules. (**B**,**C**) Data are presented as the mean ± SD, *n* = 3, *p* values were calculated using one-way ANOVA; **** *p* < 0.0001. Abbreviations: GelMA, gelatin methacrylate; CBMA, carboxybetaine methacrylate; MPC, phosphorylcholine methacrylate; SBMA, sulfobetaine methacrylate. Adapted with permission from Ref. [107].

A successful reduction in protein adsorption was demonstrated in experiments on sulfobetaine modification of silica NPs [79,95] and 4-(4-hydroxymethyl-3-methoxyphenoxy)-butyric acid (HMPB) NPs loaded with anticancer drugs [102]. The latter NPs had a high targeting capability and biocompatibility. The carboxybetaine coating of silica NPs reduced protein adsorption by as much as 94%, depending on the conjugation method [101]. Magnetic gold NPs coated with zwitterionic polymers adsorbed significantly less protein than those coated with PEG, polyethylenimine (PEI), and polyallylamine hydrochloride [105]. Modification of core/shell Fe_3_O_4_@SiO_2_ NPs with PEI and zwitterionic 2-methacryloyloxyethyl phosphorylcholine suppressed protein adsorption in a BSA solution and FBS [98]. Phosphorylcholine methacrylate (MPC) coating reduced protein adsorption on gold NPs in serum and bronchoalveolar lavage fluid (BALF) and increased their cellular uptake rate by A549 cells after treatment with these biological fluids [106]. The amount of BSA adsorbed on poly(glycidyl methacrylate) (PGMA) microspheres was noticeably decreased after the modification of their surface with the PMPDSAH poly-zwitterion [108]. Coating core/shell gelatin methacrylate MPs with carboxybetaine methacrylate (CBMA), sulfobetaine methacrylate (SBMA), and MPC strongly suppressed the nonspecific protein adsorption of the MPs, their cell adhesion, and the in vitro inflammation response to them [107] (Figure 5). The zwitterion-coated MPs implanted in mice resisted the body’s response for as long as four months.

The rates of adsorption of basic and acidic proteins on silica NPs coated with a carboxybetaine copolymer were shown to depend on the degree of the copolymer quaternization [103]. A comparison of the protein coronas formed on silica NPs, poly(2-methacryloyloxyethyl phosphorylcholine) (PMPC)-coated hybrid silica NPs, and PMPC replica particles upon incubation in human serum showed that the bare silica NPs adsorbed a wider range of proteins than the zwitterion-coated particles [109,110]. Differences in the adsorption profile between PMCP-coated and bare silica particles were confirmed in another study [36], with PMPC-coated particles forming only a “soft” corona, i.e., a layer of loosely bound proteins.

However, the adsorption of negatively charged proteins (BSA and fibrinogen) on PCL–(N-(3-sulfopropyl-N-methacryloxyethy-N,N-diethylammonium betaine) (PCL–PDEAPS) particles was more intense than on PCL–PEG particles, whereas the adsorption of lysozyme was relatively low in both cases [104].

The combination of a zwitterionic ligand with tetraethylene glycol for the functionalization of a nanodiamond surface resulted in a high colloidal stability and the suppression of protein adsorption [100].

In summary, various monomeric and polymeric zwitterionic coatings of NPs and MPs significantly decrease the protein adsorption rate. However, some drawbacks of this approach compared with PEG coating have been reported, such as a lower resistance to protein adsorption and the hindering of the interaction between surface functional groups and living cells.

### 4.3. Surface Modification with Proteins

One of the most common proteins used for surface precoating is serum albumin (BSA or HSA). The formation of the BSA corona on poly-3-hydroxybutyrate-co-3-hydroxyhexanoate (PHBHHx) NPs inhibited the adsorption of IgG on the particles, which reduced complement activation in response to their injection, prolonged their blood circulation time, and decreased their cytotoxicity [111]. A study on polymersomes with single-protein coronas showed an increased viability of cells taking up particles preliminarily incubated with IgG, lysozyme, and BSA [18].

The effect of BSA precoating on the cellular uptake of particles seems to depend on the cell line used in the study. BSA coating increased the uptake of gelatin–oleic acid NPs by HEK293 cells but decreased their uptake by A459 cells in a cell culture medium containing FBS [121].

Albumin pre-adsorption on LDH NPs enhanced their uptake by Chinese hamster ovary (CHO) cells [120]. A preformed corona of BSA modified with the RGDyK cyclic peptide on si-VEGF-loaded chitosan-based NPs reduced the protein adsorption from serum and enhanced the capacity for targeting cancer cells and efficiencies of the delivery to the tumor and suppression of angiogenesis in vivo [122,123]. The binding of sialyl Lewis A-targeted PLGA and chitosan–PLGA particles with endothelial cells in the presence of plasma was improved after coating with HSA [124]. SDS-PAGE analysis of proteins adsorbed on HSA-coated particles showed higher intensities of bands with molecular weights of 75 and 150 kDa, probably corresponding to histidine-rich glycoprotein and IgG.

Precoating with FBS weakened NP interaction with leukocytes more effectively than precoating with BSA or treatment with bovine serum, the effect correlating with a lower total protein adsorption rate and abundance of immunoglobulins [113]. BSA coating of PS NPs inhibited protein adsorption more strongly than modification with PEG or chitosan [124].

Precoating of silica NPs with γ-globulin and HSA as an opsonin and a dysopsonin, respectively, was used to modify the protein corona composition [118]. The HSA coating reduced the adsorption of coagulation factors, e.g., fibrinogen, which could enhance immune response, whereas coating with γ-globulins increased the adsorption of immunoglobulins and complement factors. However, coating with γ-globulins did not promote macrophage uptake of the NPs via Fc-mediated phagocytosis, probably because immunoglobulins were shielded with other adsorbed proteins.

Precoating with a single protein, such as BSA, myoglobin, β-lactoglobulin, lysozyme, or fibrinogen, on PS particles also affected the composition of the protein corona after incubation with serum proteins [117]. The protein of the primary corona was still detectable after incubation with serum proteins, which evidenced the stability of the protein precoating. The enhancing effect of lysozyme precoating on particle–cell interactions was shown for 200 nm PS particles, but not for 3 µm ones. Casein precoating was found to significantly decrease the amount of protein adsorbed on starch-coated poly(methyl methacrylate-co-acrylic acid) NPs; accordingly, casein-coated NPs conjugated with an aptamer exhibited a high delivery efficiency in vitro and in vivo [116].

The pre-adsorption of antibodies on PS and HES NPs formed stable targeting moieties unaffected by incubation in human serum or plasma [114]. In addition, it was shown that the targeting antibodies on the surface of polymeric NPs favored the activation of the classical complement pathway, in contrast to untargeted NPs, which activated the alternative pathway [118]. Regarding complement activation, the use of complement factor H for particle coating reduced complement activation by silica NPs [129], in accordance with previous studies postulating the stealth effect of this protein [138,139].

**Figure 6:**
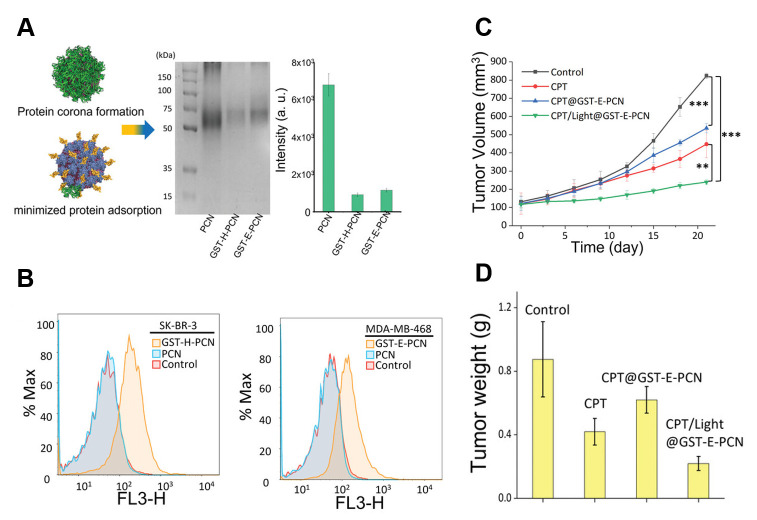
Effect of protein precoating on the targeted delivery of metal–organic framework nanoparticles (PCNs). (**A**) Gel electrophoresis of the PCNs after 1-h incubation with 50% serum solutions and the resulting band intensities of PCN-adsorbed serum proteins. (**B**) Flow cytometry analysis of the cellular uptake of DiI-loaded particles after incubation with SK-BR-3 and MDA-MB-468 cells in 50% serum media for 3 h. (**C**) Changes in tumor volume for three weeks; ** *p* < 0.01, *** *p* < 0.001, Student’s *t* test. (**D**) Weight of tumors extracted from mice after treatment with different agents. Abbreviations: CPT, camptothecin; GST, glutathione transferase; GST-H-PCNs/GST-E-PCNs, NPs coated with GST–affibody (HER2/EGFR-targeting); PCNs, metal–organic framework nanoparticles. Adapted with permission from Ref. [126].

The preliminary formation of a protein corona consisting of glutathione transferase fused with an affibody on metal–organic framework NPs led to noticeably reduced protein adsorption on them and effective targeting in vitro and in vivo [126] (Figure 6). The combination of this approach with collagenase coating enabled the deep tissue penetration of NPs and a targeted effect on tumor cells [127].

Although PEGylation and zwitterionic coating are most frequently used for preventing protein adsorption, they may decrease the blood circulation time and deteriorate the targeting capability of NPs [140]. On the other hand, the protein precoating of particles or the modification of their surface to ensure adsorption of specific proteins from blood can increase the circulation time and improve biodistribution and targeting [141].

Lipid nanocarriers consisting of 1,2-dioleoyl-3-trimethylammonium propane and DNA adsorbed vitronectin upon incubation with human plasma, which enhanced their uptake by cancer cells expressing the integrin receptor [128].

Apolipoproteins are another family of proteins used for NP and MP precoating that are known to noticeably affect particle–cell interaction. For example, precoating of PS-COOH nanoparticles with ApoA4 or ApoC3 weakened their cellular uptake, whereas coating with ApoH enhanced this process [23]. Surface coating of PS and HES NPs with clusterin (ApoJ) before incubation with IgG-enriched plasma prevented preferential adsorption of immunoglobulins, which reduced the subsequent cellular internalization [130].

Another type of protein coronas preliminarily formed on NPs and MPs before their contact with biological fluids includes the coronas consisting of protein fractions of human plasma [112]. It was found that preincubation of PS and PS-NH_2_ particles with IgG- and HSA-depleted plasma fractions prevented the aggregation of NPs placed into plasma. Preliminary coating with an HSA-containing plasma fraction facilitated the cellular uptake of NPs. The preincubation of PS particles with IgG-depleted plasma before their addition to macrophages (RAW264.7) in a plasma-supplemented cell culture significantly decreased their cellular uptake [115].

## 5. Other Factors Affecting Protein Adsorption

### 5.1. Protein Type

It is obvious that the physicochemical characteristics of proteins, such as their molecular weight, isoelectric point, and hydrophobicity, affect protein adsorption as much as the characteristics of the particles do. Therefore, the type of protein used in a study should be taken into account in interpreting the results. The protein conformation (globular or fibrillar) is a critical parameter affecting protein–particle interaction.

Most studies use BSA, HSA, fibrinogen, and immunoglobulin G as model proteins (Table 1). One of the approaches to revealing the influence of the protein properties on its adsorption is the modification of the native protein by attaching functional groups [142]. HSA with additional carboxylic or amino groups has been shown to differ from the native protein in the affinity to negatively charged quantum dots used as model NPs [142]. These results demonstrate the importance of the local charge of the protein for its interaction with NPs. On the other hand, experiments with three proteins differing in size and charge (BSA, IgG, and lysozyme) showed that all of them could be adsorbed on negatively charged polymersomes [18]. Experimental data demonstrated that interactions between polymersomes and proteins were mainly driven by hydrogen bonding and van der Waals interactions. The surface charge of the protein molecules and the ζ-potential of the particles, which are major factors of protein adsorption and subsequent association with cells, depend on the pH and ionic strength of the medium and the presence of other biomolecules [143]. In the medium with a pH value differing from the isoelectric point of the protein, a monolayer is formed due to repulsion between identical protein globules [144]. Of special interest is the possible inhomogeneity of the protein surface charge distribution, which can lead to the formation of complexes between similarly charged particles and proteins or between identically charged protein globules [145,146]. Interaction between proteins and particles is driven by various forces, including van der Waals force, hydrogen bonding, electrostatic interaction, and hydrophobic interaction, with electrostatic forces playing the main role [57].

Regarding the hydrophilicity of proteins, two different modes of adsorption on nanomicelles were demonstrated [147]. The hydrophobic bovine hemoglobin interacted with nanomicelles in the insertion mode, and the hydrophilic BSA and lysozyme, in the surface adsorption mode, with surface charge determining the differences in behavior between these two hydrophilic proteins.

### 5.2. Incubation Medium and In Vivo Studies

In order to better understand the interaction of NPs and MPs with biomolecules in the complex physiological environment, derivatives of biological fluids, such as blood serum and plasma, are commonly used in protein corona studies. A study of protein adsorption on PLGA and PLA NPs using serum and plasma as incubation media showed only minor differences in the profile and quantity of adsorbed proteins [65]. However, noticeable differences between the protein adsorption profiles of copolymer methacrylate beads incubated in serum and plasma were demonstrated [55]. In the case of PS-NH_2_ particles, the compositions of protein coronas formed upon incubation in human serum and plasma differed substantially, which was mainly accounted for by a high rate of fibrinogen adsorption in plasma [148]. The compositions of the protein coronas on silica NPs incubated with serum and plasma differed from each other, with greater amounts of coagulation and complement system proteins adsorbed from plasma than from serum [149]. The NPs incubated with plasma were also more readily taken up by RAW264.7 macrophages, but not by NIH 3T3 fibroblasts. Moreover, this study demonstrated that the particle–cell interactions after the formation of the protein corona were strongly affected by the anticoagulant heparin.

These data show that serum is advantageous as an experimental incubation medium due to the absence of coagulation proteins, whereas the advantage of plasma is that it better mimics the physiological conditions. In most cases, the compositions of protein coronas formed on particles incubated with serum and plasma differ, which usually results in differences in their cellular uptake.

An approach to the more accurate mimicking of physiological conditions is using either regular or cell-conditioned incubation media [150]. Particles whose protein coronas have formed in these two types of media differed in their macrophage uptake and stimulation of cytokine secretion. In addition, the influence of the type of washing medium on the composition of the protein corona formed on magnetic NPs was shown [29]. The composition of the NP protein corona formed in FBS was also found to depend on the medium in which the NPs were dispersed before incubation [28].

The very important study [38] demonstrated that intravenously infused liposomes can scavenge the blood pool and surface-capture low-molecular-weight, low-abundance plasma proteins that cannot be detected by conventional plasma proteomic analysis. This study also described the previously elusive or postulated formation of a protein corona around nanoparticles in vivo in humans and illustrated that it can potentially be used as a novel tool to analyze the blood circulation proteome [38]. Also, liposomes incubated in either static or dynamic (mimicking blood flow) FBS were found to differ in the protein corona composition, which further confirmed the importance of more accurately modeling in vivo conditions [151]. An in vitro study of the interaction of individual proteins with NPs estimated the binding affinities of albumin, transferrin, fetuin A, and hemoglobin to NPs with different surface charges and the potential changes in the secondary structure of the proteins [152]. These results correlated with earlier data on the effect of proteins adsorbed on NPs in vivo on their blood circulation time [153]. However, a comparison of the protein corona formed on lipid NPs in vivo 10 min after intravenous injection with that formed in vitro showed significant quantitative and qualitative differences in protein composition [154]. Another example is the considerable differences in the protein coronas formed on PS NPs modified with transferrin-receptor-targeting ligands that were incubated under in vitro and in vivo conditions [155]. Fibrinogen was one of the most abundant proteins in the corona formed in vitro, whereas it accounted for less than 1% of the in vivo corona. In contrast, the amounts of adsorbed albumin and clusterin were higher in the in vivo corona. Similarly, the protein composition of the corona formed on magnetic NPs in vivo was more diverse than that of the in vitro corona [156]. In order to better mimic in vivo conditions, the authors of that study suggested an ex vivo approach, with NPs incubated in freshly isolated blood for 1 min. In this case, the composition of the protein corona was similar to that of the in vivo corona. Thus, the results of in vitro and in vivo studies of protein adsorption on particles differ in terms of protein corona composition, relative amounts of individual proteins, and targeting efficiency. These discrepancies between the protein coronas formed on NPs in vitro and in vivo hamper the clinical translation of nanotherapeutics.

In summary, protein adsorption on NPs and MPs is strongly affected by experimental conditions, including the incubation medium and overall experimental design. To better understand the protein corona formation and its biological consequences, it is necessary to accurately select optimal experimental conditions closely mimicking physiological conditions.

## 6. Effect of Protein Adsorption on Particle–Cell Interactions

### 6.1. Targeted Delivery

**Figure 7:**
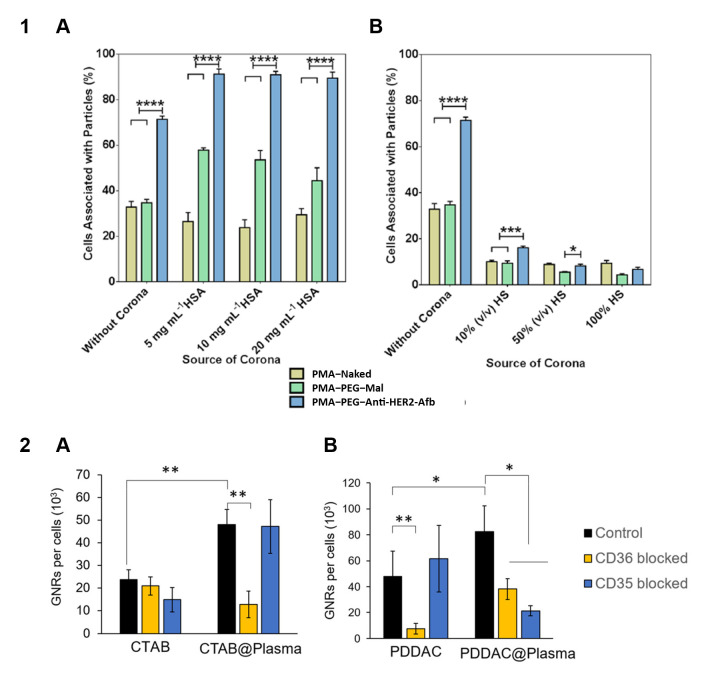
Effect of protein adsorption on particle–cell interactions. **Panel 1.** Association of affibody-functionalized polymer particles with SK-OV-3 cells after the formation of protein coronas from (**A**) human serum albumin solutions and (**B**) media containing human serum at different concentrations. * *p* < 0.05; *** *p* < 0.001; **** *p* < 0.0001. Adapted with permission from Ref. [159]. **Panel 2.** Internalization of GNRs with surface modifications by (**A**) CTAB and (**B**) PDDAC, quantified by inductively coupled plasma mass spectrometry under normal conditions and after the blocking of CD35 and CD36 receptors with the respective antibodies. The number of GNRs internalized per cell was calculated and is presented as the mean ± SD, *n* = 4. The statistical differences with significance are marked as * *p* < 0.05, ** *p* < 0.01. Abbreviations: GNRs, gold nanorods; CTAB, cetyltrimethylammonium bromide; PDDAC, poly(diallyldimethyl ammonium chloride). Adapted with permission from Ref. [167].

The influence of protein adsorption on the behavior of engineered NPs and MPs used for targeted delivery is crucial for the development of effective delivery systems. The binding of transferrin-functionalized NPs with both the soluble transferrin receptor and A549 cells expressing this receptor was decreased after adsorption of proteins [157]. The targeting capacity of NPs functionalized with transferrin was lost after the formation of the protein corona in vitro but partly retained after its formation in vivo [158]. Note that transferrin–NPs preserved the targeting specificity towards brain tumor cells after crossing the blood–brain barrier. Protein adsorption from human serum significantly suppressed the interaction of affibody-functionalized, polymer-coated silica particles with SK-OV-3 cells, whereas adsorption of human serum albumin enhanced the specificity of this interaction [159] (Figure 7, Panel 1). This effect of protein adsorption on the specific binding of particles can be explained by the shielding of ligands by adsorbed proteins, which prevents them from interacting with the receptor.

However, polymeric particles and capsules conjugated with monoclonal antibodies were reported to retain their targeting ability after protein adsorption [53]. The effect of protein adsorption on the interaction of hyaluronic acid (HA)-modified metal–phenolic network (MPN) capsules depended on the molecular weight of HA: the capsules modified with 13 kDa HA lost their targeting ability, whereas those modified with 230 kDa HA exhibited only a 5% reduction in specific binding [160]. In the case of Fn14-targeted NPs, the reduction in cellular uptake after incubation with serum was greater in NPs functionalized with the Fab fragment than in those functionalized with the full-length antibody [161].

### 6.2. Cellular Uptake

Considerable differences in the cellular uptake of silica NPs were observed in serum-free and complete culture media [162]. In the serum-containing media, a protein corona that decreased the adhesion and internalization of the particles by A549 cells was formed. Preincubation of polymeric NPs with plasma also reduced their uptake by Telo-RF and HeLa cells [163]. The formation of a protein corona on lipid and silica NPs reduced their uptake by RAW 264.7 macrophages [164]. The adsorption of proteins on polymer particles in an FBS-supplemented cell medium had different effects on the association of the particles with THP-1 monocytes and with macrophage-like cells differentiated from them [165]. Particles with a BSA-rich protein corona were internalized by THP-1 monocytes less efficiently than bare particles. On the other hand, the rate of particle internalization by the differentiated macrophage-like cells remained unchanged after the adsorption of proteins on the particles.

The presence of adsorbed proteins on the particle surface can affect the cell entry mechanism. After the incubation of liposomes with human plasma and the formation of a protein corona containing a large portion of apolipoproteins, HeLa cells mainly took up the liposomes via clathrin-mediated endocytosis [166]. In the case of gold nanorods modified with cetyltrimethylammonium bromide (CTAB) or poly-diallyldimethylammonium chloride (PDDAC), the mechanisms of particle internalization by macrophages were different for plasma-incubated and pristine nanorods. The uptake of plasma-incubated nanorods was reduced if the cells were pretreated with anti-CD36 antibodies, which indicated the involvement of the CD36 scavenger receptor, probably due to the presence of apolipoproteins and transport proteins on the nanorod surface [167] (Figure 7, Panel 2). Different mechanisms underlie the cellular uptake of NPs whose protein coronas are formed in media with high and low concentrations of serum, which was explained by differences in the corona composition [168]. Experiments with silica NPs showed that the presence of apolipoprotein B on the NP surface determined NP recognition by the LDL receptor, whereas surface IgG was recognized by the Fc-γ receptor I [169].

The secondary structure of the adsorbed proteins was also shown to determine the type of receptors that bind protein-coated NPs [170]. In the case of anionic NPs, adsorbed BSA retained its native conformation, which allowed the binding of the NP–BSA complexes by albumin receptors. In contrast, adsorption of BSA on cationic NPs led to BSA denaturation and the subsequent binding of the NP–BSA complexes by scavenger receptors.

There are data on the roles of individual proteins adsorbed on the particle surface in modulating cellular uptake. For example, PS NP coating with ApoA4 and ApoC3 considerably reduced the uptake of these NPs by human mesenchymal stem cells, whereas coating with ApoH enhanced the cellular uptake [23]. A negative correlation between ApoA4 adsorption on mesoporous silica–PEG NPs and their association with cells (monocytes and B cells) was reported [74].

The protein coronas of NPs and MPs can facilitate their use as vehicles for targeted drug delivery. For example, an apolipoprotein-rich corona formed on lipid NPs ensured their targeted delivery to HepG2 tumor cells [27]. Clusterin (ApoJ) was identified as the key component of a protein corona providing the stealth effect (reduction in nonspecific cellular uptake) of PEGylated PS NPs [80] and unmodified silver and silica nanocarriers [171].

The presence of immunoglobulins and complement proteins (C4B and C3) on the particle surface was found to facilitate the association of particles with immune cells (monocytes and B cells) [74]. For example, the adsorption of C1q on NPs promoted opsonization with the C3 component and subsequent recognition by leukocytes and macrophages [172]. The binding of IgG to the surface of different nanomedicines was shown to induce the deposition of the C3 component [173]. The adsorption of plasma proteins on mesoporous silicon and PMPC particles proved to be required for their association with blood cells (monocytes, granulocytes, and B cells) [110]. Precoating with IgG promoted the association of particles with phagocytic cells. The enrichment of the protein corona with complement proteins was more pronounced on mesoporous silicon particles than on PMPC ones and was positively correlated with their association with B cells.

**Figure 8:**
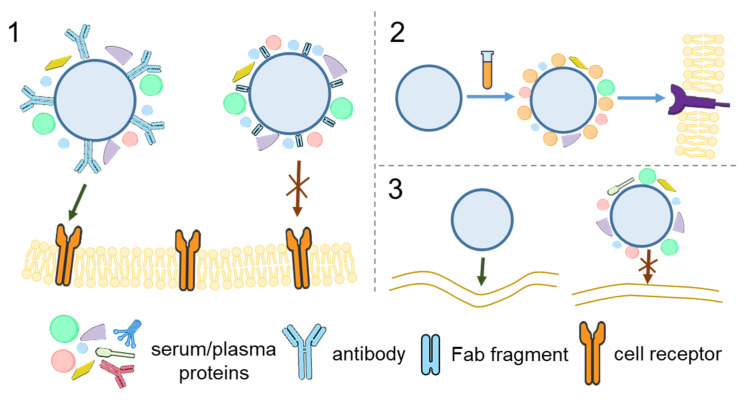
The main mechanisms of protein adsorption influence on (**1**,**2**) targeted particle delivery to cells and (**3**) cellular uptake.

Thus, protein adsorption is a crucial factor that influences particle–cell interactions in protein-containing media. The binding of particles to cells after protein adsorption can be either enhanced or suppressed, depending on the composition of the protein corona, cell type, and particle uptake mechanism. It is necessary to evaluate the possible effects of protein corona formation on particles designed for targeted delivery to cells. These data could be used both to avoid undesirable effects of protein adsorption on cell targeting and to facilitate targeting through specific protein–receptor interactions. The main mechanisms of the influence of protein adsorption on particle–cell interactions are summarized in Figure 8.

## 7. Conclusions and Outlook

NPs and MPs are extensively used in the development of new biosensors, imaging agents, and theranostic tools. More than 20 delivery carriers based on lipid and polymeric particles have been approved for clinical use in the past 15 years [174]. When NPs and MPs enter biological fluids, they are coated with a protein corona, whose structure, size, and composition strongly depend on the morphological and physicochemical properties of the particle surface. The intricate composition of the protein corona coating NPs and MPs is determined by the complexity of the surrounding biological medium and the tendency towards the minimization of the surface energy of the particles through interactions with protein molecules. In turn, the protein corona affects the biodistribution of the particles, including their circulation time, interaction with cells, and, eventually, transport to the target site. Understanding and harnessing the complex mechanisms of protein adsorption on the engineered NPs and MPs are crucial for enhancing the efficiency of nanomedical applications while ensuring their safety. This is especially important in developing NPs and MPs as drug delivery vehicles, because the agents based on them are intended for intravenous, intranasal, or intramuscular administration, and the administration route directly determines the composition of their protein corona and their subsequent distribution in the body. The development of NPs and MPs with a desired protein corona composition may become a new step towards their targeted delivery. This will make it possible to obtain more effective drugs with reduced side effects, as well as more accurate and specific biosensors and imaging tools. Of special interest is the role of machine learning (ML) and neural networks in predicting the formation of protein corona on NPs and MPs in biological fluids and their subsequent biological interactions. For example, ML models predicting the NP protein corona composition and corona-mediated interaction of NPs with cells have been developed [175,176]. The application of computational approaches to protein corona research could give new insight into the complex interconnection between particle properties, incubation conditions, and protein adsorption parameters and promote the analysis of nano–bio interactions influenced by the protein corona. However, the development of adequate models capable of these predictions requires a huge amount of reliable standardized experimental data on the composition of the protein corona and the biodistribution of protein-coated NPs and MPs.

This review focuses on the influence of the key physicochemical and morphological parameters of NPs and MPs on the quantitative characteristics of protein adsorption on their surface. Available data show that the particle size is directly related to the amount of the adsorbed protein, with larger particles typically binding more protein. The amounts of different proteins adsorbed on particles depend to different degrees on the particle size and may affect the cellular uptake of the particles. The particles’ shape, surface morphology, and surface structure, as well as their composition, also largely determine both the total amount of the adsorbed proteins and their proportions in the protein corona.

The surface chemistry and charge of the particles are other key factors determining the interaction with proteins. In general, the protein adsorption rate increases with increasing particle surface charge. Various surface functional groups also strongly affect the formation of the protein corona. In vivo, NPs and MPs are usually taken up by cells via endocytosis, the acidic internal medium and enzymes of endosomes and lysosomes also affecting the protein corona composition and structure.

The interactions of NPs and MPs with biological media may interfere with the targeted delivery of the particles, shortening their circulation time. One of the major strategies for preventing this effect by reducing or modifying the protein adsorption is the attachment of hydrophilic polymers, such as PEG or PG, to the particle surface. The presence of hydrophilic polymers on the surface of the carriers affects not only the amount of the adsorbed proteins, but also the preferential adsorption of some of them, e.g., apolipoproteins. The modification of the surface with zwitterionic compounds is also a promising way to make the particles protein-repellent. Alternatively, the precoating of particles with some proteins allows the modulation of subsequent protein adsorption and particle–cell interactions. In some cases, modification of the particles with specific proteins enables the desired biodistribution and efficient targeting of NPs and MPs. Further development of surface modification approaches is aimed at reducing the particle toxicity and immunogenicity and modulating the protein adsorption on the particles and the biological response to them.

This review is also aimed at attracting researchers’ attention to the necessity for standardizing the conditions of protein corona research. For example, some studies have shown that there are differences in protein corona formation when NPs and MPs are incubated in biological fluids in stationary and flow phases [150], as well as when NPs and MPs of similar compositions are fabricated and/or functionalized by different methods [75]. Differences between the biological fluids used for the incubation of NPs and MPs pose another problem in analyzing the structure and composition of the protein corona, because there are data on differences between the protein coronas formed not only, e.g., upon incubation in FBS or human serum, but even upon incubation in human sera from donors of different sexes and ages or with different diseases [74]. Therefore, these factors should be taken into consideration in developing various drug delivery, biosensing, and imaging agents. In addition, the techniques and conditions of the analysis of a protein corona, including the media [28] and washing buffers used [29,31], have been shown to strongly affect the results.

An analysis of the available published data on the influence of morphological and physicochemical characteristics of NPs and MPs (Table 1), as well as their surface modifications (Table 2), on the adsorption of corona proteins has shown considerable differences both between the results of different studies and between the experimental conditions used in them. This complicates the development of neural network tools for analyzing and predicting the composition of a protein corona, as well as the biodistribution of NPs and MPs as dependent on their structure and composition. This is why we would like to highlight the issues that deserve particular attention in designing experiments to study the effects of the morphology and physicochemical properties of NPs and MPs on the formation and composition of the protein corona, as well as on their biodistribution and interaction with cells, and in reporting the results of these experiments:

- The methods for synthesizing NPs and MPs and for the functionalization of their surface should be standardized;
- The characteristics of the synthesized NPs and MPs, including their size and shape, surface morphology and porosity, density of coating with ligands, shell elasticity, hydrophilic/hydrophobic properties, and surface charge, before they come into contact with biological fluids should be estimated and described in detail;
- Proteomic profiling of the biological fluids (blood serum, saliva, etc.) used in the study should be performed;
- The conditions of the analysis, including the compositions of the buffers, incubation conditions (e.g., incubation in a flow or stationary phase or the use of a shaker) should be described;
- The kinetics of the changes in the composition of the protein corona as dependent on the incubation and washing conditions should be analyzed;
- Where possible, in addition to in vitro study of the composition of the protein corona, in vivo experiments should be planned to estimate the biodistribution of NPs and MPs and their interaction with cells.

Obtaining sets of standardized data, together with the control of input parameters and experimental conditions, will make it possible to move from studying a protein corona to predicting its composition and other characteristics, as well as the biodistribution of NPs and MPs as dependent on their properties. Overall, the understanding of factors influencing protein adsorption on particles and various approaches to its reduction or modification can improve the design of NPs and MPs for biomedical applications. One important approach to prolonging NP and MP circulation in vivo is to damp the interaction of NPs and MPs with immune cells by using sets of particles with identical cores but different shells.

In recent years, multidisciplinary studies combining materials science, physical, chemical, and biological approaches, as well as the use of advanced engineering and computational tools, have yielded numerous data on the formation and role of protein coronas. This gives grounds to expect that the coming years will bring exciting breakthrough theranostic applications making use of the fundamental properties of protein coronas reviewed in this article.

## Figures and Tables

**Table 1:** Effect of the morphological and physicochemical properties of nano- and microparticles on protein adsorption.

Type of Particles	Variable Parameter(s)	Incubation Medium	Main Conclusions	Ref.
Nonporous silica particles, 50 and 500 nm; mesoporous silica particles, 500 nm	Size, porosity	FBS	The amount of adsorbed protein directly depends on the available surface area of particles. The protein profile is independent of surface area and porosity.	[12]
Silica particles with amidine surface modification, 30–1000 nm	Size	BSA * and myoglobin * solutions	Conformational changes of myoglobin and BSA upon adsorption are size-dependent.	[49]
Polystyrene particles, 50 and 100 nm; bare, carboxyl- and amine-modified	Size, surface chemistry	Human plasma	Size and surface chemistry significantly affect the composition of the protein corona.	[21]
Polystyrene microspheres (PS MSs), 1.33 and 4.46 μm, original and with etched surface	Size, surface morphology	Human plasma, BSA */fibrinogen ** solution	Protein adsorption is lower on etched PS MSs; the lowest adsorption is on PS MSs with the maximum number and minimum area of protuberances on the surface. This effect and total adsorption depend on size. MSs after etching (which changes the surface morphology) adsorb low-molecular-weight proteins; control MSs, a wider range of proteins.	[22]
Polystyrene particles, 100–500 nm	Size	HEWL * solution	The particles form a protein corona of HEWL, mainly driven by hydrophobic interactions. The formation of amyloid fibrils by HEWL is promoted by 100, 400, and 500 nm particles.	[47]
Dense and mesoporous silica particles, 70–900 nm	Size, porosity	FBS	Both dense and porous particles with smaller size adsorb more protein; small dense particles adsorb proteins with lower Mw; all porous particles adsorb smaller proteins.	[44]
Elastin-like polypeptide (ELP)-based particles, 200 and 500 nm	Size, surface chemistry	Platelet-poor human plasma	All ELP constructs adsorb a large amount of albumin, immunoglobulin G, and activated complement factor 3; variations in the composition between different NPs are observed for plasminogen, fibronectin, activated fibrinogen, antithrombin, and alpha2 macroglobulin.	[45]
LMW chitosan oligosaccharide (COS) NPs, 130 and 150 nm, and HMW chitosan (CS) NPs, 95 and 110 nm	Size, composition	BSA * and HSA * solutions	CS NPs weakly interact with proteins because of the high structural rigidity of the polymer. The smaller CS NPs exhibit greater BSA binding.	[41]
Pyrolytic and colloidal silica particles, 20–120 and 50 nm, respectively	Size, surface chemistry	BSA * solution	The difference in protein coverage appears to be related to differences in the distribution of surface silanols more than to differences in ζ-potential.	[66]
PS, PS-COOH, and PS-NH_2_ particles, 50 nm and 1 μm	Size, surface chemistry	Human saliva	Surface chemistry and size of the particles affect the adsorption of saliva proteins. The formation of the protein corona causes changes in the surface charge, aggregation, and, hence, in vitro cytotoxicity.	[68]
PS particles, 50, 100, 200, and 500 nm and 1 μm; PS particles with the –COOH and –NH_2_ groups, 100 nm	Size, surface chemistry	Simulated salivary fluid, simulated gastric fluid, simulated intestinal fluid	The protein corona formed after in vitro digestion alters the macrophage uptake of uncharged 50 and 100 nm PS particles, but not charged 100 nm or larger particles. The presence of digestion proteins changes the adsorption of serum proteins from cell culture media.	[67]
Silica NPs, 8, 33, and 78 nm	Size	Yeast protein extract	Larger NPs adsorbed more protein per surface unit; most of the proteins adsorbed on NPs with different sizes were identical.	[46]
PEGylated gold NPs, 20–150 nm	Size	HDL solution, human serum	Larger NPs exhibit a larger surface coverage with HDLs. This process is regulated by nonspecific interactions rather than the adsorption of apolipoprotein A-I.	[47]
Solid lipid nanoparticles (SLNs), 120–480 nm	Size	BSA * solution	BSA adsorption increases with the increasing size of the particles.	[25]
PEGylated plasmonic gold NPs (rods and stars), 40 and 70 nm	Size, shape	In vivo in CD-1 mice	Gold nanostars adsorb more protein than nanorods due to their larger surface area. The composition of the protein corona varies between NPs with different sizes and shapes.	[9]
Polystyrene NPs, 26, 80, and 200 nm	Size	Mouse serum	Protein corona formation is size-dependent.	[42]
Gold NPs modified with PEG, 21–109 nm	Size	FBS	Protein adsorption is greater on larger gold NPs.	[43]
Gold NPs (spheres, rods, stars, and cages) functionalized with R-PEG-SH (R = OCH_3_, COOH, or NH_2_), ∼50 nm	Shape, surface chemistry	Human serum	Cage-shaped NPs adsorb less protein and differ in protein corona composition.	[10]
Mesoporous silica particles, spherical, faceted, and rod-shaped	Shape	Bovine serum	Considerable differences in the protein composition, surface coverage, and particle agglomeration of the protein corona–particle complex indicate specific protein adsorption profiles highly dependent on the number of exposed facets and aspect ratio.	[48]
Mesoporous silica particles: spherical, 270 nm; rod-shaped, 290 × 1100 nm	Shape	Serum, plasma	Rod-shaped particles adsorb more protein from serum and plasma.	[51]
Diethylaminoethyl-dextran cellulose microspheres, 100 μm; pore size, 165–282 nm	Pore size	BSA * and γ-globulin * solutions	Increasing-then-decreasing trends in the adsorption equilibria and uptake kinetics are observed as the pore size increases. A critical pore size at which the adsorption capacity and uptake are significantly enhanced has been determined.	[52]
Poly(methacrylic acid) (PMA) capsules and particles functionalized with mAbs	Structure	Human serum	The capsules and particles differ in the protein corona composition. The protein corona has little effect on the targeting ability of the mAb-functionalized capsules and particles.	[53]
Silica nanocapsules, 150–180 nm, with stiffnesses (Young moduli) of 704 kPa, 25 MPa, 459 MPa, and 9.7 GPa	Stiffness	FBS	The protein corona of the stiffest nanocapsules contains the largest amount of complement protein C3 and immunoglobulin proteins, which facilitates their macrophage uptake.	[58]
Hydrogel NPs with a lipid bilayer shell and unmodified or PEGylated surface, 100–150 nm; elasticity, 45 kPa–760 MPa	Stiffness	Mouse plasma	The protein corona composition varies nonmonotonically depending on the NP elasticity; the relative abundance of Apo-I is correlated with the blood clearance lifetime of the NPs.	[61]
Polymeric core/multishell, solid cationic and anionic NPs and polymeric soft nanogels (NGs), 75–175 nm	Surface chemistry	Human plasma	Solid anionic and cationic particles adsorb more protein than other particles; NPs with a protein corona more weakly interact with cells; NGs with a protein corona induce enhanced cytokine release	[20]
Core/shell and shell-only multilayered polyelectrolyte particles, 2 μm	Structure	Human serum, plasma	Differences in particle structure affect both the quantity of adsorbed proteins and the relative abundance of individual protein groups (apolipoproteins, complement proteins, and immunoglobulins).	[54]
PLGA-NPs, 152 nm; PLGA/cholesterol NPs, 237 nm; cholesterol NPs, 257 nm	Composition	Human plasma	Different protein corona compositions on particles with different matrix compositions in the presence or absence of a targeting ligand have been shown.	[15]
MAA and PMMA polymeric beads	Composition, incubation medium	Serum, plasma	The two materials differ with respect to protein adsorption, which is associated with differences in complement activation.	[55]
Sulfated alginate, high G alginate, and poly-L-lysine-coated alginate microspheres, 565, 579, and 538 μm, respectively	Composition	Lepuridin-anticoagulated plasma	Differences in the adsorption of complement and coagulation proteins can explain differences in the inflammatory and fibrotic responses to three types of alginate microspheres.	[56]
Lipid nanodiscoids, 47–63 nm	Composition	Mouse serum	The phospholipid composition affects the protein corona composition, which determines the particle stability, circulation, and biodistribution.	[26]
Elastomer (star-poly(D,L-lactide-co-e-caprolactone)) E2 and E5 microspheres, 184 and 370 μm	Composition	FBS, human plasma	Polymer chain flexibility affects protein adsorption in competitive adsorption environments.	[60]
Lipid NPs, 67–110 nm	Composition	Nude mice serum	Differences in lipid constituents altered the composition of the protein corona; NPs with an apolipoprotein-rich corona showed higher delivery efficiency than NPs with a vitronectin-rich corona.	[27]
PLGA and PCL particles, 416 and 559 nm	Composition	HSA *, human serum	Serum proteins have a higher affinity to more hydrophobic (PCL) particles; the protein corona composition is material-specific.	[16]
Poly(methyl methacrylate-*co*-styrene) particles, ∼550 nm	Composition	BSA *, IgG *, and fibronectin * solutions	Protein adsorption is enhanced on particles with a higher amount of styrene due to a higher hydrophobicity.	[19]
Albumin, cationic albumin, and ovalbumin cross-linked NPs, 165–206 nm	Composition	FBS	Protein-based NPs with various hydrophobicities and charges differ in the protein corona pattern; the protein corona affects in vitro macrophage uptake of BSA and cationic BSA NPs but not ovalbumin NPs.	[24]
Polystyrene NPs with –COOH, –NH_2_, –SO_3_, and –PO_3_ surface groups, ~100 nm	Surface chemistry	Human serum	High enrichment of apolipoproteins on NPs and preferential binding of apolipoproteins on NH_2_- and SO_3_-functionalized NPs have been found.	[23]
Poly(styrene) surfaces modified with –NH_2_, –COOH, and –PO_3_H_2_	Surface chemistry	FBS	Protein adsorption varied between surfaces with different surface groups; this was related to differences in their binding by macrophages and inflammatory responses to them.	[61]
PLA and PLGA NPs, 188–224 nm	Surface chemistry, hydrophobicity, matrix polymer Mw, incubation medium	Human serum, plasma	The PLA matrix polymer Mw affects only the types of the bound proteins; the adsorption of specific proteins depends on hydrophobicity; surface end group identity influences both the total amount and the profile of the adsorbed proteins.	[65]
Glycosylated polyhydroxy polymer-modified nanovesicles (CP-LVs), ~100 nm	Surface chemistry	Plasma, liver tissue interstitial fluid	CP-LVs with the highest amino-to-hydroxyl ratio are characterized by decreased immunoglobulin adsorption and prolonged blood circulation; the adsorption of tumor-specific proteins leads to effective cellular internalization.	[63]
Polyelectrolyte-doped block copolymer-stabilized NPs, 40–180 nm	Surface chemistry	BSA * solution	NPs with an anionic (PAA) surface modification tend to adsorb less protein and are taken up only by macrophage-like cells, whereas cationic (DMAEMA) NPs are taken up by different types of cells.	[64]

*—globular protein; **—fibrillar protein. Abbreviations: BSA, bovine serum albumin; COS, chitosan oligosaccharide; CP-LVs, glycosylated polyhydroxy polymer-modified nanovesicles; CS, chitosan; DMAEMA, poly(N,N-dimethylaminoethyl methacrylate); ELP, elastin-like polypeptide; FBS, fetal bovine serum; HDL, high-density lipoprotein; HEWL, hen egg-white lysozyme; MAA, methacrylic acid co-methyl methacrylate; mAb, monoclonal antibody; NG, nanogel; NP, nanoparticle; PAA, poly(acrylic acid); PCL, polycaprolactone; PEG, poly(ethylene glycol); PLA, poly(lactic acid); PLGA, poly(lactic-co-glycolic) acid; PMA, poly(methacrylic acid); PMMA, polymethyl methacrylate; PS, polystyrene; SLNs, solid lipid nanoparticles.

**Table 2:** Surface modifications of nano- and microparticles and their effect on protein adsorption.

Type of Particles	Modification	Incubation Medium	Main Conclusions	Ref.
Ovalbumin NCs, 250–300 nm	PEG	Human plasma	Confirmation of PEG affects the adsorption of specific proteins (dysopsonins) on NCs and their uptake by phagocytic cells.	[69]
Polystyrene NPs, 90–100 nm	Lutensol AT50 (PEG-based surfactant), SDS	HSA solution	The NPs coated with Lutensol adsorb more protein than those coated with SDS; however, the loss of conformation is less pronounced in PEGylated (Lutensol-modified) NPs.	[70]
Phosphonic acid-terminated poly(ethylene glycol)-conjugated layered double hydroxide (PEG-LDH) NPs, 100–170 nm	PEG	BSA solution	PEGylation of LDH particles enhances their stability, reduces BSA adsorption, and enhances cellular uptake.	[71]
PEGylated gold NPs (GNPs), 125–214 nm	PEG (350, 550, 1000 Da)	Human plasma	GNPs modified with PEG 550 adsorb more albumin and transferrin, which correlates with higher uptake by, and cytotoxicity for, HepG2 cells and a higher antitumor effect in mice.	[72]
Mesoporous silica particles (MSNs), 980 nm	PEG (2000, 5000, 10,000 Da)	BSA solution	The depth of BSA penetration into MSNs is higher than that of MSNs-PEG; the maximum penetration depth decreases with increasing PEG chain length.	[73]
Mesoporous silica (MS), MS-PEG, and PEG particles, 100, 450, and 800 nm	PEG	Human plasma	The rate of immune cell association of MS-PEG particles with protein coronas formed from plasmas of individual donors widely varies depending on the donor; the cell association of all PEG particles is minor, irrespective of the protein corona source.	[74]
Mesoporous silica NPs (MSNs), 210–250 nm	PEG	10% FCS in PBS	The method of PEGylation affects the NP pore size and the amount of adsorbed proteins.	[75]
Gold NPs (AuNPs), 18–78 nm	PEG (5000, 10,000, 30,000 Da)	BSA, GB3 (WT and K19C), and H1.5 peptide (with and without cysteine) solutions	The amount of adsorbed peptides/proteins decreases with decreasing PEG size; small molecules (glutathione, H1.5 peptide, GB3) can penetrate PEG layers; the presence of thiol groups enhances the adsorption on PEGylated AuNPs.	[76]
Strontium sulfite NPs (SSNs), 670–2100 nm	PEG	10% mouse plasma	PEGylation of SSNs leads to a reduction in protein adsorption from serum, a reduction in cytotoxicity, and efficient tumor delivery.	[77]
PLGA and PLGA-PEG NPs, 100 and 200 nm	PEG	FBS	PEGylation significantly decreases the amount of bound proteins.	[78]
SiO_2_, SiO_2_–sulfobetaine silane (SBS), and SiO_2_-PEG_2000_ NPs, ~100 nm	Zwitterion, PEG	BSA and lysozyme solutions	Functionalization of SiO_2_ NPs with PEG and SBS prevents BSA adsorption but not lysozyme adsorption, although the latter is reduced, with no particle aggregation.	[79]
Polystyrene particles with –NH_2_, –PEG, or poly(ethyl ethylene phosphate) (PEEP) groups, 100–120 nm	PEG, PEEP	Human plasma	In addition to reducing protein adsorption, PEG and PEEP can affect the composition of the protein corona, and the presence of specific proteins is necessary to prevent nonspecific cellular uptake.	[80]
Alginate/chitosan/alginate (ACA) hydrogel microcapsules modified with chitosan–methoxy poly-(ethylene glycol) (CS-*g*-MPEG), 350 μm	MPEG	IgG solution	The adsorption of IgG is drastically reduced on microcapsules coated with CS-*g*-MPEG. The protein repulsion capacity depends on the degree of substitution and chain length of MPEG.	[81]
Hydroxyethyl starch NPs functionalized with PEG and modified with mannose	PEG, PEG–mannose	Human plasma	The PEG and PEG–mannose modifications of HES NPs reduce the protein adsorption but do not affect the protein corona composition. HES–PEG–mannose NPs are efficiently bound by dendritic cells.	[82]
Hydroxyethyl starch NPs functionalized with PEG	PEG	Human serum	PEGylation of HES NPs reduces the protein adsorption from serum by 37%.	[83]
Gold NPs functionalized with fluorescent Herceptin and backfilled with methoxy-terminated PEG (mPEG), 73–97 nm	PEG (1000, 2000, 5000, 10,000 Da)	Human serum	NPs with attached PEG groups adsorb fewer proteins in the presence of serum but retain the targeting specificity.	[84]
Iron oxide nanoparticles (IONPs) coated with polyethylene glycol–allyl glycidyl ether copolymer functionalized with RGD peptide or transferrin, 22–31 nm	PEG-*b*-AGE	FBS, human plasma	The coating of the NPs with PEG-*b*-AGE leads to a decrease in protein adsorption and nonspecific uptake by macrophages of NPs, irrespective of functionalization.	[85]
PEGylated liposomal drug (Onyvide), 60 nm	PEG	Human plasma	PEGylation of liposomes does not prevent the formation of the protein corona; adsorbed proteins from plasma enhance binding to PANC-1 cells.	[86]
Gold NPs modified with PEG, 21–109 nm	PEG	FBS	PEG modification reduces plasma protein adsorption.	[43]
Liposomes functionalized with PEG or hyperbranched polyglycerol, 60–90 nm	PEG, PG	Human plasma	Protein adsorption on unmodified and PEG- and PG-modified liposomes is relatively low; macrophage uptake of liposomes is not affected by the protein corona; modification with PEG reduces cellular uptake, whereas modification with PG enhances it.	[87]
Nanodiamonds (NDs), 30, 50, and 100 nm; SPIONs, 22 nm	PEG, PG	FBS, human plasma	PG modification more efficiently reduces the protein adsorption and macrophage uptake than PEG modification.	[88]
Polystyrene NPs modified with poly(phosphoester) surfactants, ∼110 nm	PPE	HSA, plasma	Noncovalent modification of PS NPs with poly(phosphoester) surfactants reduces the protein adsorption and macrophage uptake after incubation in plasma.	[89]
Poly(organosiloxane) NPs functionalized with PEG or poly(2-ethyl-2-oxazoline), 22–26 nm	PEG, PEtOx	Bovine serum	Modification of the NPs with either PEG or PEtOx reduces the protein adsorption; modification with PEtOx reduces the cellular uptake in both serum and serum-free medium.	[90]
Biotin- and cysteine-coated silica NPs, 120 nm	Zwitterion (amino acid)	10, 50, 100% human plasma	Zwitterionic (cysteine) coating interferes with the protein corona formation and improves the NP targeting ability.	[91]
Silica NPs functionalized with lysine/arginine/glycine/cysteine/phenylalanine, ∼60 nm	Zwitterions (amino acids)	BSA, 10% FBS	Functionalization of NPs with cysteine, lysine, and arginine reduces protein adsorption, the effect of lysine being the strongest.	[92]
Silica NPs (SiO_2_-organosiloxane), ∼120 nm	Zwitterion	BSA and lysozyme solutions, 50% FBS	Zwitterated silica NPs display antifouling properties in both BSA and lysozyme solutions, as well as in FBS.	[93]
Gold NPs conjugated with zwitterionic peptides or PEG, 50–100 nm	Zwitterions (peptides)	Human serum	Protein adsorption is reduced up to 60% compared with bare NPs; the protein adsorption profile depends on the charge motif and sequence but not amino acid composition.	[94]
Bi-functionalized silica NPs (SiO_2_–organosiloxane/COOH), ∼140 nm	Zwitterion	BSA and lysozyme solutions, 50% FBS	SiO_2_–organosiloxane/COOH_5/1_ particles absorb less protein while retaining the capacity for effectively binding target biomolecules.	[95]
Sulfobetaine-coated silica NPs, 250 nm	Zwitterion	BSA solution, FBS	Zwitterion modification of silica NPs results in a 90% decrease in protein adsorption rate and a reduced NP uptake by macrophages and fibroblasts.	[96]
Polystyrene/Fe_3_O_4_/silica NPs functionalized with amino/alkene/betaine, 230–440 nm	Zwitterion	BSA and lysozyme solutions, FBS	Betaine functionalization reduces the protein adsorption from single-protein solutions and serum, with amino and alkene groups remaining accessible for interaction with biomolecules.	[97]
PEI-coated core/shell Fe_3_O_4_@SiO_2_ NPs functionalized with 2-methacryloyloxyethyl phosphorylcholine (MPC), ∼125 nm	Zwitterion	BSA solution, FBS	The protein corona formation in 50-MPC- and 75-MPC-functionalized silica NPs is reduced in BSA solution and FBS.	[98]
Silica NPs functionalized with zwitterion and amino/mercapto/carboxylic groups, ∼100 nm	Zwitterion	BSA solution	Double-functionalized NPs exhibited a low protein adsorption level, with biologically active groups shielded from interactions.	[99]
Nanodiamonds (NDs) functionalized with zwitterion and tetraethylene glycol (TEG), ∼100 nm	Zwitterion	FBS	Combined surface functionalization of NDs with TEG chains and zwitterionic head groups ensures colloidal stability and prevents protein adsorption.	[100]
Silica NPs functionalized with carboxybetaine, 40 nm	Zwitterion	BSA solution	Protein binding to functionalized NPs is significantly reduced (by up to 94%).	[101]
Hollow mesoporous Prussian blue NPs (HMPBs) functionalized with sulfobetaine and peptide E5, 130 nm	Zwitterion	BSA solution	HMPBs@PEI-ZW and HMPBs@PEI-ZW-E5 NPs adsorb noticeably less BSA than the original NPs.	[102]
Poly(DMAEMA-co-carboxybetaine methacrylate)-modified silica NPs (MCB), 137–220 nm	Zwitterionic copolymer	BSA and lysozyme solutions	Modification of silica NPs using carboxybetaine copolymer with different quaternization degrees modulates the adsorption of basic and acidic proteins.	[103]
Poly(ε-caprolactone)-*b*-poly(N,N-diethylaminoethyl methacrylate)/(N-(3- sulfopropyl-N-methacryloxyethy-N,N-diethylammonium betaine)) (PCL-PDEAPS) and poly(ε-caprolactone)-*b*-poly(ethylene glycol) (PCL-PEG) micelles, 77–109 and 51 nm, respectively	Zwitterionic polymers, PEG	BSA, lysozyme, and fibrinogen solutions; plasma	PCL-PDEAPS micelles adsorb more negatively charged proteins (BSA, fibrinogen) than PCL-PEG micelles due to electrostatic interactions; PCL-PDEAMS micelle uptake by macrophages after protein adsorption is low.	[104]
Magnetic gold NPs functionalized with poly(carboxybetaine methacrylate) and poly 2-carboxy- N,N-dimethyl-N-(2′-(methacryloyloxy)ethyl)ethanaminium), 123 nm	Zwitterion	HSA solution, serum	Magnetic gold NPs with CBMA coating adsorb significantly less protein than control NPs coated with PEG, PAH, or PEI.	[105]
Gold NPs coated with thiol-functionalized polymethacryloyloxyethyl phosphorylcholine polymers (pMPC), 230 nm	Zwitterion	Human serum, BALF	pMPC-coated gold NPs exhibit reduced protein adsorption and increased uptake by A549 cells after exposure to serum.	[106]
Core/shell GelMA microcapsules; zwitterionic coated CBMA-GelMA, SBMA-GelMA, and MPC-GelMA microcapsules, ~600 μm	Zwitterionic polymers	FITC-BSA in PBS	Zwitterionic coating effectively blocks nonspecific protein adsorption.	[107]
Poly(glycidyl methacrylate) (PGMA) microspheres modified with [3-(methacryloylamino)propyl]dimethyl(3-sulfopropyl)ammonium hydroxide (MPDSAH), 2 μm	Zwitterionic polymers	BSA solution	The amount of adsorbed BSA was reduced to up to half of that on the unmodified PGMA microspheres.	[108]
Silica particles with poly(2-methacryloyloxyethyl phosphorylcholine) coating	Zwitterionic polymers	HSA solution	Coating with zwitterions prevents the adsorption of tightly bound proteins, but a “soft” protein corona forms on the particles.	[36]
Mesoporous silica particles, poly(2-methacryloyloxyethylphosphorylcholine) (PMPC)-coated silica hybrid particles, PMPC replica particles, 164, 161, and 179 nm, respectively	Zwitterionic polymers	Human serum, human blood	Mesoporous silica particles adsorb more proteins of a wider range of molecular weights than PMPC-coated and PMPC replica particles.	[109]
Mesoporous silica particles and poly(2-methacryloyloxyethyl phosphorylcholine) (PMPC) particles, with or without coating with HSA, IgG, and C1q, ∼1 µm	Zwitterionic polymers, proteins	Human plasma, whole blood, washed blood	PMPC particles exhibit reduced adsorption of serum proteins and association with cells. The enrichment of the corona with some proteins facilitates association with specific cell types.	[110]
Poly(acrylic acid)-*block*-polystyrene (PAA_22_-*b*-PS_144_) polymersomes, 60 nm	BSA, IgG, lysozyme	–	The formation of protein coronas from all model proteins used reduces the cytotoxicity of the polymersomes.	[18]
Poly-3-hydroxybutyrate-co-3-hydroxyhexanoate (PHBHHx) NPs, ~200 nm	BSA	Rat serum	The preformed albumin corona inhibits the plasma proteins’ adsorption, reduces the complement activation, prolongs the blood circulation time, and reduces the toxicity of the PHBHHx NPs.	[111]
PS particles with –COOH and –NH_2_ groups or without functionalization, 58–69 nm	Human plasma fractions (IgG, HSA, and low-abundant protein)	Human plasma	Preincubation with IgG-, HSA-, and low-abundant-protein-enriched plasma fractions alters the cellular uptake and inhibits the aggregation of PS and PS-NH_2_ particles in human plasma.	[112]
Supramolecular template NPs, metal–phenolic network (MPN)-coated template (core/shell) NPs, and MPN nanocapsules, ~100 nm	BSA, FBS, bovine serum	Human blood	Precoating using FBS effectively reduces the amount of adsorbed proteins and alters the composition of the protein corona, which reduces NP association with leukocytes.	[113]
PS particles with –COOH and –NH_2_ groups, 150–230 nm; hydroxyethyl starch (HES) NPs, 450 nm	Antibodies	Human serum or plasma	NPs with pre-adsorbed antibodies retain the targeting ability after the formation of the biomolecular corona.	[114]
PS particles with –COOH and –NH_2_ groups, 130–150 nm	IgG-depleted plasma	Cell culture medium containing human plasma	Precoating of NPs with Ig-depleted plasma reduces their cellular uptake; the preformed protein corona remains stable after reintroduction to plasma.	[115]
Starch-coated poly(methyl methacrylate-co-acrylic acid) nanoparticles, 85 nm	Casein, soybean protein isolate, myoglobin, zein, gelatin	30% FBS	NPs precoated with casein exhibit antifouling properties and excellent targeting in vitro and in vivo.	[116]
PS particles, 0.2 and 3 μm	BSA, myoglobin, β-lactoglobulin, lysozyme, fibrinogen	10% FCS in cell culture medium	Precoating of particles strongly affects protein corona formation; primary proteins can still be detected after the second incubation. Precoating affects particle–cell interactions, but not particle toxicity.	[117]
Carboxylic acid-functionalized silica NPs, ~100 nm	γ-globulin, HSA	10 or 55% human plasma in PBS	Precoating of the NPs with γ-globulins, but not HSA, leads to the enrichment of the protein corona with immunoglobulins and complement factors, but the opsonin-rich corona does not enhance NP uptake by macrophages.	[118]
Polylactic acid (PLA)–PEG–poly(ε-caprolactone) (PCL) NPs, 118 and 189 nm	Antibody	Mouse serum or human serum	Complement proteins are adsorbed on the NP surface. NPs bearing the targeting antibody activate the classical immune pathway; untargeted NPs, the alternative pathway.	[119]
Layered double hydroxide (LDH) NPs, ~100 nm	BSA	Cell culture medium	BSA precoating stabilizes LDH suspensions in cell culture medium and enhances NP uptake by CHO cells.	[120]
Gelatin–oleic nanoparticles (GONs)	BSA	Cell culture medium containing FBS	The uptake of GONs precoated with BSA by HEK293 cells is enhanced, but their uptake by A549 cells is suppressed in a FBS-containing cell medium.	[121]
Chitosan-based nanocarriers (TsR NPs) loaded with siVEGF, 500–1500 nm	BSA-cRGD	10% FBS	The adsorption of BSA-cRGD on TsR NPs results in reduced serum protein adsorption and improves tumor targeting and therapeutic efficiency.	[122]
siVEGF-loaded chitosan-based NPs (CsR/siVEGF NPs), 150–1000 nm	BSA-cRGD	10% mouse serum, 10% FBS	Precoating of CsR/siVEGF NPs with BSA-cRGD enhances their stability in serum and tumor targeting efficiency, which is related to reduced protein adsorption from serum and changes in the protein corona composition.	[123]
Polystyrene NPs coated with PEG, BSA, chitosan, and cell membranes from a human breast cancer cell line, 110–150 nm	PEG, BSA, chitosan, cell membranes	10% FBS	All the tested coatings reduce protein adsorption, the effects of the BSA and cell membrane coatings being the strongest.	[124]
PLGA and chitosan–PLGA microparticles, ∼1.6 μm	HSA	Human plasma	HSA coating changes the protein profile of the corona and enhances binding to endothelial cells.	[125]
Zr_6_-based metal–organic framework NPs (PCN-224), 180–260 nm	GST-Afb	50% serum	Precoating of PCN-224 with a protein layer reduces the protein adsorption on NPs and improves cancer cell and tumor targeting.	[126]
Metal–organic framework particles (MOF-808) loaded with camptothecin, 90–120 nm	GST-Afb and collagenase	50% serum	The formation of a protein layer consisting of GST-Afb and collagenase reduces protein adsorption from serum and provides efficient tissue penetration and tumor targeting.	[127]
DOTAP/DNA lipid particles, ∼220 nm	Human plasma	–	Spontaneous coating with vitronectin promotes uptake by cancer cells overexpressing α_ν_β_3_ integrin, the vitronectin receptor.	[128]
Silica NPs, 110 nm	BSA, HSA, IgG, complement factor H, fibrinogen	Human serum	Coating with BSA, HSA, and fibrinogen reduces the adsorption of complement proteins on the NP surface; coating with complement factor H inhibits complement activation by the NPs.	[129]
Polystyrene NPs, 50 nm; HES NPs, 110 nm	Clusterin (apolipoprotein J)	IgG-enriched human plasma	Precoating of both types of NPs with clusterin reduces IgG adsorption from IgG-enriched plasma and suppresses cellular uptake.	[130]
Polystyrene NPs (PS-COOH), 100 nm	Apolipoproteins ApoA4, ApoC3, and ApoH; prothrombin; antithrombin III	–	Coating with ApoA4 and ApoC3 reduces NP uptake by hMSCs, whereas coating with ApoH enhances uptake. Prothrombin and antithrombin III do not affect cellular uptake significantly.	[23]

Abbreviations: BSA, bovine serum albumin; CBMA, carboxybetaine methacrylate; cRGD, cyclo(Arg-Gly-Asp-d-Tyr-Lys-) peptide; CS, chitosan; DMAEMA, poly(N,N-dimethylaminoethyl methacrylate); DOTAP, 1,2-dioleoyl-3- trimethylammonium propane; FBS, fetal bovine serum; FCS, fetal calf serum; FITC, fluorescein isothiocyanate; GB3, third IgG-binding domain of protein G; GNPs, gold nanoparticles; GONs, gelatin–oleic nanoparticles; GST-Afb, glutathione transferase–affibody; HES, hydroxyethyl starch; HMPBs, hollow mesoporous Prussian blue nanoparticles; HSA, human serum albumin; IONPs, iron oxide nanoparticles; LDH, layered double hydroxide; MPC, 2-methacryloyloxylethyl phosphorylcholine; mPEG, methoxy poly(ethylene glycol); MPN, metal–phenolic network; MSNs, mesoporous silica nanoparticles; NC, nanocarrier; ND, nanodiamond; NP, nanoparticle; PCL, polycaprolactone; PDEAPS, N-(3-sulfopropyl-N-methacryloxyethy-N,N-diethylammonium betaine); PEEP, poly(ethyl ethylene phosphate); PEG, poly(ethylene glycol); PEG-*b*-AGE, poly(ethylene glycol) and allyl glycidyl ether block copolymer; PEtOx, poly(2-ethyl-2-oxazoline); PG, poly(glycerol); PGMA, poly(glycidyl methacrylate); PHBHHx, poly-3-hydroxybutyrate-co-3-hydroxyhexanoate; PLA, poly(lactic acid); PLGA, poly(lactic-co-glycolic) acid; PPE, poly(phosphoester); PS, polystyrene; SBMA, sulfobetaine methacrylate; SBS, sulfobetaine silane; SDS, sodium dodecyl sulfate; siVEGF, small interfering RNA targeting vascular endothelial growth factor; SPIONs, superparamagnetic iron oxide nanoparticles; TEG, tetraethylene glycol; ZW, zwitterion.

## Data Availability

No primary research results, software, or code has been included, and no new data other than those presented in this publication were generated or analyzed as part of this review.

## Abbreviations

The following abbreviations are used in this manuscript:

NP	Nanoparticle
MP	Microparticle
PLGA	Poly(lactic-co-glycolic acid)
PS	Polystyrene
PEG	Polyethylene glycol
BSA	Bovine serum protein
ELP	Elastin-like polypeptide
HDL	High-density lipoprotein
MAA	Methacrylic acid co-methyl methacrylate
PMMA	Polymethyl methacrylate
PCL	Poly(ε-caprolactone)
ApoE	Apolipoprotein E
FBS	Fetal blood serum
PS-*b*-PEG	Poly(styrene)-block-poly(ethylene glycol)
LDH	Layered double hydroxide
HES	Hydroxyethyl starch
PEG-*b*-AGE	Allyl glycidyl ether
PEEP	Poly(ethyl ethylene phosphate)
ApoJ	Apolipoprotein J
HMPB	4-(4-hydroxymethyl-3-methoxyphenoxy)-butyric acid
PEI	Polyethylenimine
MPC	Phosphorylcholine methacrylate
BALF	Bronchoalveolar lavage fluid
PGMA	Poly(glycidyl methacrylate)
CBMA	Carboxybetaine methacrylate
SBMA	Sulfobetaine methacrylate
PMPC	Poly(2-methacryloyloxyethyl phosphorylcholine)
PCL–PDEAPS	PCL–(N-(3-sulfopropyl-N-methacryloxyethy-N,N-diethylammonium betaine)
PHBHHx	Poly-3-hydroxybutyrate-co-3-hydroxyhexanoate
CHO	Chinese hamster ovary
CTAB	Cetyltrimethylammonium bromide
PDDAC	Poly-diallyldimethylammonium chloride
